# D-Cysteine Ethyl Ester Reverses the Deleterious Effects of Morphine on Breathing and Arterial Blood–Gas Chemistry in Freely-Moving Rats

**DOI:** 10.3389/fphar.2022.883329

**Published:** 2022-06-23

**Authors:** Paulina M. Getsy, Santhosh M. Baby, Walter J. May, Alex P. Young, Benjamin Gaston, Matthew R. Hodges, Hubert V. Forster, James N. Bates, Christopher G. Wilson, Tristan H. J. Lewis, Yee-Hee Hsieh, Stephen J. Lewis

**Affiliations:** ^1^ Department of Pediatrics, Case Western Reserve University, Cleveland, OH, United States; ^2^ Department of Drug Discovery, Galleon Pharmaceuticals, Inc., Horsham, PA, United States; ^3^ Pediatric Respiratory Medicine, University of Virginia School of Medicine, Charlottesville, VA, United States; ^4^ Herman B Wells Center for Pediatric Research, Indiana University School of Medicine, Indianapolis, IN, United States; ^5^ Department of Physiology, Medical College of Wisconsin, Milwaukee, WI, United States; ^6^ Department of Anesthesia, University of Iowa Hospitals and Clinics, Iowa City, IA, United States; ^7^ Basic Sciences, Division of Physiology, School of Medicine, Loma Linda University, Loma Linda, CA, United States; ^8^ Division of Pulmonary, Critical Care and Sleep Medicine, University Hospitals Case Medical Center, Case Western Reserve University, Cleveland, OH, United States; ^9^ Department of Pharmacology, Case Western Reserve University, Cleveland, OH, United States

**Keywords:** D-cysteine ethyl ester, thiol esters, morphine, respiratory depression, gas exchange, antinociception, sedation, Sprague Dawley rats

## Abstract

Cell-penetrant thiol esters including the disulfides, D-cystine diethyl ester and D-cystine dimethyl ester, and the monosulfide, L-glutathione ethyl ester, prevent and/or reverse the deleterious effects of opioids, such as morphine and fentanyl, on breathing and gas exchange within the lungs of unanesthetized/unrestrained rats without diminishing the antinociceptive or sedative effects of opioids. We describe here the effects of the monosulfide thiol ester, D-cysteine ethyl ester (D-CYSee), on intravenous morphine-induced changes in ventilatory parameters, arterial blood–gas chemistry, alveolar–arterial (A-a) gradient (i.e., index of gas exchange in the lungs), and sedation and antinociception in freely-moving rats. The bolus injection of morphine (10 mg/kg, IV) elicited deleterious effects on breathing, including depression of tidal volume, minute ventilation, peak inspiratory flow, and inspiratory drive. Subsequent injections of D-CYSee (2 × 500 μmol/kg, IV, given 15 min apart) elicited an immediate and sustained reversal of these effects of morphine. Morphine (10 mg/kg, IV) also A-a gradient, which caused a mismatch in ventilation perfusion within the lungs, and elicited pronounced changes in arterial blood–gas chemistry, including pronounced decreases in arterial blood pH, pO_2_ and sO_2_, and equally pronounced increases in pCO_2_ (all responses indicative of decreased ventilatory drive). These deleterious effects of morphine were immediately reversed by the injection of a single dose of D-CYSee (500 μmol/kg, IV). Importantly, the sedation and antinociception elicited by morphine (10 mg/kg, IV) were minimally affected by D-CYSee (500 μmol/kg, IV). In contrast, none of the effects of morphine were affected by administration of the parent thiol, D-cysteine (1 or 2 doses of 500 μmol/kg, IV). Taken together, these data suggest that D-CYSee may exert its beneficial effects via entry into cells that mediate the deleterious effects of opioids on breathing and gas exchange. Whether D-CYSee acts as a respiratory stimulant or counteracts the inhibitory actions of µ-opioid receptor activation remains to be determined. In conclusion, D-CYSee and related thiol esters may have clinical potential for the reversal of the adverse effects of opioids on breathing and gas exchange, while largely sparing antinociception and sedation.

## Introduction

Opioids, such as morphine and fentanyl, are administered to alleviate pain. However, their clinical utilization is complicated by their propensity to adversely affect breathing and ventilatory mechanics (van Dorp et al., 2007; Dahan et al., 2010; Boom et al., 2012; Dahan et al., 2018; Algera et al., 2019). Opioid receptor (OR) antagonists, such as naloxone, cannot only prevent and reverse opioid-induced respiratory depression (OIRD), but also reverse opioid-induced analgesia and sedation. Therefore, their clinical value is limited in many situations, including during surgery and in post-surgical situations where pain relief and sedation are essential to patients (Dahan et al., 2010; Dahan et al., 2018). Accordingly, there is an urgent unmet need to develop drugs that reverse OIRD by mechanisms other than antagonism of ORs. In a recent comprehensive review, Dahan et al. (2018) concluded that no currently available experimental drugs are adequate for the therapeutic reversal of OIRD for a host of reasons, including that they only minimally improve arterial oxygenation, and they all need to be further evaluated for their true efficacy and for potential adverse (i.e., toxicological) effects.

Our recent studies have detailed the ability of reduced (monothiol) and oxidized (disulfide) thiol esters to prevent/reverse the adverse effects of opioids, such as morphine and fentanyl, on ventilatory parameters and arterial blood–gas (ABG) chemistry in rats without compromising the analgesic or sedative actions of the opioids (Mendoza et al., 2013; Gaston et al., 2021; Jenkins et al., 2021). These studies were based on evidence that morphine inhibits L-cysteine uptake into neurons via blockade of excitatory amino acid transporter type 3 (Trivedi et al., 2014; Trivedi and Deth, 2015), and substantial evidence that systemically administered ethyl ester and methyl ester derivatives of reduced and oxidized thiols readily enter cells including cells in the periphery, and neurons in the central nervous system (Servin et al., 1988; Foreman and Benson, 1990; Lailey et al., 1991; Ben-Nun et al., 1992; Hobbs et al., 1993; Lailey and Upshall, 1994; Chu et al., 2004; Rech et al., 2008; Figueiredo et al., 2009; Ito et al., 2012; Mendoza et al., 2013; Sumayao et al., 2013; Gurbuz et al., 2015; Henderson et al., 2016).

We began our investigations with L-cysteine ethyl ester (L-CYSee), and reported that the bolus intravenous injection of L-CYSee produced a prompt and sustained reversal of the adverse effects of morphine on ABG chemistry and alveolar–arterial (A-a) gradient (i.e., an index of alveolar gas exchange) in isoflurane-anesthetized tracheotomized rats, but not in rats without the tracheal tube (Mendoza et al., 2013). The finding that L-cysteine was inactive suggested that the actions of L-CYSee were due to entry into neurons/cells, and the initiation of intracellular signaling events that may involve redox-dependent processes. With respect to therapeutic potential, it was evident that the ability of L-CYSee to overcome the adverse actions of morphine on breathing and A-a gradient is overridden by adverse effects on upper airways, which may include the collapse of the larynx and vocal folds, and/or the loss of muscle activity in the tongue with the flaccid tongue blocking the airway (Mendoza et al., 2013). We are currently examining whether ethyl ester and methyl ester derivatives of D-cysteine and D-cystine can reverse OIRD without the adverse effects of the L-thiol esters with the expectation that (1) D-thiol esters rapidly enter cells/neurons as efficiently as L-thiol esters; (2) the adverse effects of the L-thiol esters involve them entering into metabolic pathways, while D-thiol esters do not; and (3) the specific (as yet undefined) intracellular processes by which thiol esters reverse OIRD are not dependent upon stereospecific processes. Our first report with a D-isomer demonstrated that the intravenous administration of the disulfide esters, D-cystine dimethyl ester or D-cystine diethyl ester, reversed the adverse effects of morphine on ventilatory parameters and ABG chemistry in freely-moving rats without adversely affecting the antinociceptive actions of the opioid (Gaston et al., 2021).

This study extends our understanding of the pharmacological profiles of D-thiol esters by presenting the effects of D-cysteine ethyl ester (D-CYSee) and the parent thiol, D-cysteine, on the pharmacological actions of morphine in unanesthetized/unrestrained adult male Sprague Dawley rats. We report here that the intravenous injection of D-CYSee elicits a rapid and sustained reversal of the adverse effects of morphine on ventilatory parameters, A-a gradient, and ABG chemistry with minimal effects on the analgesic or sedative actions of the opioid, whereas D-cysteine was inactive. This profile of activity of D-CYSee would be advantageous in clinical settings in which opioids are essential for pain relief, yet their adverse effects on ventilation need to be overcome.

## Materials and Methods

### Permissions, Rats, and Surgical Procedures

All studies were carried out in accordance with the NIH Guide for the Care and Use of Laboratory Animals (NIH Publication No. 80-23) revised in 1996, and in strict compliance with the ARRIVE (Animal Research: Reporting of *In Vivo* Experiments) guidelines (http://www.nc3rs.org.uk/page.asp?id=1357). All experimental protocols were approved by the Animal Care and Use Committees of Case Western Reserve University, the University of Virginia, and Loma Linda University.

Adult male Sprague Dawley rats were purchased from Harlan Industries (Madison, WI, United States). After four to five days of recovery from transportation, the rats were implanted with femoral artery catheters and/or jugular vein catheters under 2% isoflurane anesthesia as detailed previously (Henderson et al., 2014; Gaston et al., 2021). The rats were given at least 4 days to recover from surgery before use. All femoral arterial catheters were flushed daily with heparin solution (50 units of heparin in 0.1 M, pH 7.4, phosphate-buffered saline). On the day of experimentation, all arterial and venous catheters were flushed with phosphate-buffered saline (0.1 M, pH 7.4) approximately 4 hours before the commencement of experiments. Note that the pH values of all stock solutions of the test drugs (i.e., vehicle, D-cysteine and D-CYSee) were adjusted to approximately 7.2 with 0.25M NaOH. All studies were performed in a quiet laboratory with a relative humidity of 50 ± 2% and room temperature of 21.3 ± 0.2°C. The antinociception and ventilatory/ABG chemistry experiments were performed in separate groups of rats so as to not compromise the respiratory measurements. The plethysmography, antinociception recording sessions, and the arterial blood sampling studies (ABG assays) were conducted by the same investigator who injected the opioid, vehicle, or test drugs (i.e., D-cysteine and D-CYSee). The syringes containing the vehicle or test drugs were made up by another investigator, such that the person running the actual experiment was blinded to the treatment protocol. In every case, the data files resulting from each study were collated and then analyzed by yet another investigator in the group.

### Protocols for Whole Body Plethysmography Measurement of Ventilatory Parameters

Ventilatory parameters were recorded continuously in the unrestrained freely-moving rats using a whole body plethysmography system (PLY3223; Data Sciences International, St. Paul, MN) as detailed previously (May et al., 2013a; May et al., 2013b; Young et al., 2013; Getsy et al., 2014, 2020; Henderson et al., 2014; Baby et al., 2018, 2021a,b; Gaston et al., 2020, 2021; Seckler et al., 2021). The directly recorded and calculated (derived) parameters are defined in Supplementary Table S1 (Hamelmann et al., 1997; Lomask, 2006; Tsumuro et al., 2006; Quindry et al., 2016; Gaston et al., 2021). Supplementary Figure S1 shows a diagram of relationships between some of the directly recorded parameters (adapted from Lomask, 2006). On the day of the study, each rat was placed in an individual plethysmography chamber and allowed at least 60 min to acclimatize so that the resting (baseline (pre)) ventilatory parameter values are accurately defined.

Study 1: Two groups of rats received a bolus injection of morphine (10 mg/kg, IV), and after 15 min, one group (*n* = 5, 77.4 ± 0.4 days of age, and 331 ± 2 g body weight) received a bolus injection of vehicle (saline) and the other group (n = 6, 77.6 ± 0.4 days of age, and 333 ± 2 g body weight) received a bolus injection of D-CYSee (500 μmol/kg, IV). The rats then received a second injection of either vehicle or D-CYSee 15 min later depending on their group. Study 2: Two groups of rats received a bolus injection of morphine (10 mg/kg, IV), and after 15 min, one group (*n* = 5; 77.9 ± 0.3 days of age; and 329 ± 2 g body weight) received a bolus injection of vehicle (saline) and the other group (*n* = 6; 77.8 ± 0.4 days of age; and 330 ± 2 g body weight) received a bolus injection of D-cysteine (500 μmol/kg, IV). The rats then received a second injection of either vehicle or D-cysteine 15 min later depending on their group. Ventilatory parameters were monitored for 60 min after the second set of vehicle/D-CYSee/D-cysteine injections in all groups of rats. We gave two injections of D-CYSee to see if the first injection produces an effect, and if not, whether it is needed to prime the effect of the second injection. Study 3: One group of rats (*n* = 6; 81.6 ± 0.6 days of age; and 345 ± 3 g body weight) received a bolus injection of morphine (10 mg/kg, IV), and after 15 min, they received a bolus injection of D-CYSee (500 μmol/kg, IV), and after another 15 min, they received a bolus injection of vehicle rather than D-CYSee. These rats are referred to as the D-CYSee (1x) group in Figures 5, 6. Whereas another group of rats received two bolus injections of D-CYSee 15 min apart, and they are referred to as the D-CYSee (2x) group. Note that the resting ventilatory parameters of the D-CYSee (1x) rats were similar to those of the D-CYSee (2x) rats (*p* < 0.05, for all comparisons *via* an unpaired *t*-test, data not shown).

The body weights of all the groups of rats were similar to one another, thus corrections of ventilatory parameters (e.g., TV, PIF, and PEF) are shown without correcting for body weight. The FinePointe (DSI) software constantly corrected digitized ventilatory values originating from the actual waveforms for alterations in chamber humidity and temperature. Pressure changes associated with the respiratory waveforms were then converted to volumes (e.g., TV, PIF, PEF, and EF_50_) by employing the algorithms of Epstein et al. (Epstein and Epstein, 1978; Epstein et al., 1980). Specifically, factoring in chamber temperature and humidity, the cycle analyzers filtered the acquired signals, and FinePointe algorithms generated an array of box flow data that identified a waveform segment as an acceptable breath. From this array, the minimum and maximum box flow values were determined and multiplied by a compensation factor provided by the selected algorithm (Epstein and Epstein, 1978; Epstein et al., 1980), thus producing TV, PIF, and PEF values that were used to determine non-eupneic breathing events expressed as the non-eupneic breathing index (NEBI), reported as the percentage of non-eupneic breathing events per epoch (Getsy et al., 2014). The apneic pause was determined by the formula, (Expiratory Time/Relaxation Time) − 1 (Gaston et al., 2021).

### Protocols for Blood–Gas Measurements and Determination of Arterial–Alveolar Gradient

Alterations in key ABG chemistry parameters—pH, pCO_2_, pO_2_ and sO_2_—elicited by the bolus injection of morphine (10 mg/kg, IV) in three different sets of unanesthetized freely-moving rats (*n* = 9 rats per group) followed 15 min later by a bolus injection of vehicle (saline, IV; 81.3 ± 0.8 days of age; and 343 ± 2 g body weight), D-cysteine (500 μmol/kg, IV; 82.5 ± 0.5 days of age; and 347 ± 3 g body weight), or D-CYSee (500 μmol/kg, IV; 81.4 ± 0.4 days of age; 341 ± 2 g body weight) were determined as detailed previously (Henderson et al., 2014; Baby et al., 2018, Baby et al., 2021 S.; Gaston et al., 2021; Jenkins et al., 2021). Samples of arterial blood (100 μl) were taken 15 min before and 15 min after the injection of morphine (10 mg/kg, IV). The rats then immediately received an injection of vehicle, D-cysteine or D-CYSee, and blood samples were taken at 5-, 15-, 30-, and 45-min time points. The pH, pCO_2_, pO_2_ and sO_2_ were determined with the aid of a radiometer blood–gas analyzer (ABL800 FLEX). The A-a gradient measures the difference between alveolar and arterial blood O_2_ concentrations (Stein et al., 1995; Story 1996; Henderson et al., 2014). A reduction in PaO_2_ without a concomitant alteration in A-a gradient is the result of hypoventilation, whereas a reduction in PaO_2_ with a concomitant elevation in A-a gradient is an indicator of an ongoing mismatch in ventilation–perfusion in the lungs (Stein et al., 1995; Story 1996; Henderson et al., 2014; Gaston et al., 2021). A-a gradient = PAO_2_ − PaO_2_, where PAO_2_ is the partial pressure (*p*) of alveolar O_2_, and PaO_2_ is pO_2_ in the sampled arterial blood. PAO_2_ = [(FiO_2_ x (P_atm_ − P_H2O_) − (PaCO_2_/respiratory   quotient)], where FiO_2_ is the fraction of O_2_ in inspired air, P_atm_ is the atmospheric pressure, P_H2O_ is the partial pressure of H_2_O in inspired air, PaCO_2_ is pCO_2_ in arterial blood, and respiratory quotient (RQ) is the ratio of CO_2_ eliminated to O_2_ consumed. We took FiO_2_ of room air to be 21% = 0.21, P_atm_ to be 760 mmHg, and P_H2O_ to be 47 mmHg (Gaston et al., 2021). We took the RQ value of our adult male rats to be 0.9 (Stengel et al., 2010; Chapman et al., 2012; Gaston et al., 2021; Jenkins et al., 2021).

### Antinociception Assessment by Tail-Flick Latency Assay

The antinociceptive effects elicited by an injection of morphine and a subsequent injection of vehicle or D-CYSee were determined by evaluating tail-flick latency (TFL) with the use of a Tail-Flick Analgesia Meter (IITC Life Science Inc., USA) as described previously (Lewis et al., 1991; Meller et al., 1991; Carstens and Wilson, 1993; Henderson et al., 2014; Golder et al., 2015; Gaston et al., 2021; Jenkins et al., 2021). This procedure entailed a minor level of manual restraint during the positioning of the tail to apply a thermal stimulus sufficient to induce a latency of tail withdrawal of about 3.0 s in all rats. Baseline TFL was tested in all rats prior to any drug administration (−20-min time point). One group of rats (*n* = 6; 78.3 ± 1.4 days of age; and 332 ± 3 g body weight) received an injection of morphine at 5 mg/kg (IV), and after TFL testing at + 20 min, they immediately received a bolus IV injection of vehicle (saline, 100 μl/100 g body weight). A second group of rats (*n* = 6; 79.5 ± 1.5 days of age; and 335 ± 3 g body weight) received an injection of morphine (5 mg/kg, IV), and after TFL testing at + 20 min, they immediately received a bolus injection of D-CYSee (500 μmol/kg, IV). A third group (*n* = 6; 77.3 ± 2.2 days of age; and 336 ± 4 g body weight) received an injection of morphine at 10 mg/kg (IV), and after TFL testing at + 20 min, they immediately received a bolus IV injection of vehicle (saline, 100 μl/100 g body weight). A fourth group (*n* = 6; 78.0 ± 1.8 days of age; and 334 ± 2 g body weight) received an injection of morphine (10 mg/kg, IV), and after TFL testing at + 20 min, they immediately received a bolus injection of D-CYSee (500 μmol/kg, IV). TFL was tested at +20, 40, 60, 90, 120, 180 min time points. The resulting data are presented as actual TFL (sec) and as maximum possible effect (%MPE) determined by the formula, %MPE = [(post-injection TFL − baseline TFL)/(12 − baseline TFL)] x 100 (Lewis et al., 1991; Meller et al., 1991; Carstens and Wilson, 1993; Henderson et al., 2014; Golder et al., 2015; Gaston et al., 2021; Jenkins et al., 2021).

### Sedation as Determined by the Modified Righting Reflex Test

Adult male Sprague Dawley rats were used to evaluate the effects of bolus injections of vehicle, D-cysteine (500 μmol/kg, IV) and D-CYSee (500 μmol/kg, IV), on the duration of morphine (10 mg/kg, IV)-induced impairment of the modified righting reflex test. Each rat was placed in an open plastic chamber to allow the duration of the loss of the modified righting reflex to be accurately determined. In essence, the injection of morphine resulted in the rats assuming a variety of distinct postures, including being sprawled out motionless on their stomach on the chamber floor, splayed out with the head up against the chamber wall, and lying motionless on their side. The duration of the sedative effects of morphine was determined by the time interval from the time of injection of morphine to full recovery of the modified righting reflex, taken when the rats assumed and kept a normal posture on all four legs (Ren et al., 2015, 2020; Yu et al., 2018; Jenkins et al., 2021). One set of rats (*n* = 12; 79.7 ± 0.6 days of age; 330 ± 2 g body weight) received a bolus injection of morphine (10 mg/kg, IV) and after 15 min, an IV injection of vehicle (saline). A second set of rats (*n* = 12; 80.1 ± 0.6 days of age; and 335 ± 3 g body weight) received a bolus injection of morphine (10 mg/kg, IV) and after 15 min, an injection of D-cysteine (500 μmol/kg, IV). A third set of rats (*n* = 12; 80.1 ± 0.6 days of age; and 335 ± 3 g body weight) received a bolus injection of morphine (10 mg/kg, IV) and after 15 min, an injection of D-CYSee (500 μmol/kg, IV).

### Data Analyses

All data are presented as mean ± SEM and were evaluated using one-way and two-way ANOVAs followed by Bonferroni’s corrections for multiple comparisons between means using the error mean square terms from each ANOVA (Wallenstein et al., 1980; Ludbrook, 1998; McHugh, 2011) as described in detail previously (Getsy et al., 2021). A value of *p* < 0.05 was taken as the initial level of statistical significance (Wallenstein et al., 1980; Ludbrook, 1998; McHugh, 2011). Statistical analyses were performed using GraphPad Prism software (GraphPad Software, Inc., La Jolla, CA, United States).

## Results

### Effects of D-CYSee and D-Cysteine on Ventilatory Responses to Morphine

**FIGURE 1:**
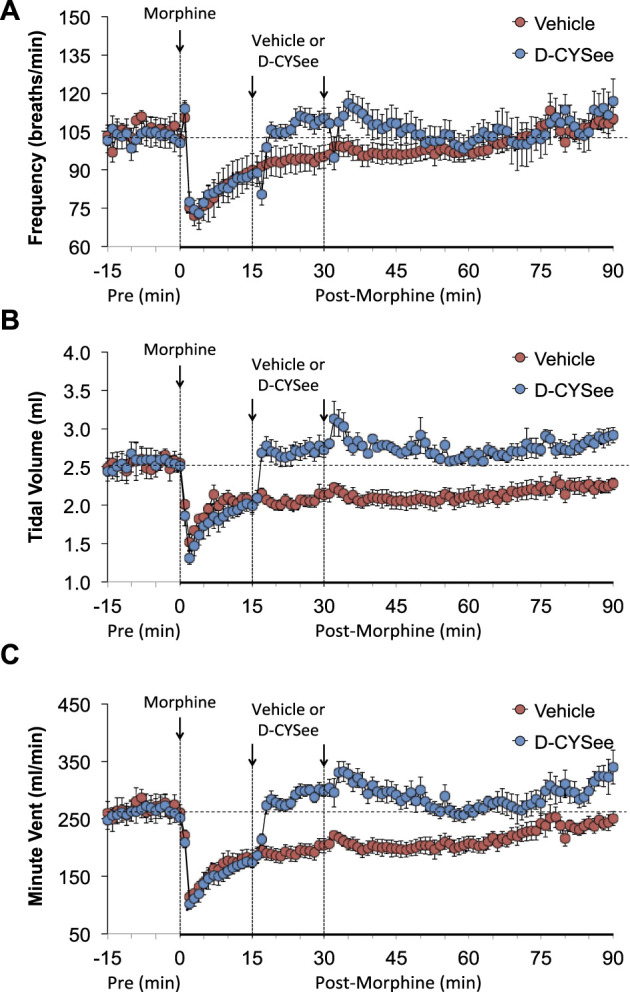
Values of frequency of breathing (Panel **(A)**), tidal volume (Panel **(B)**), and minute ventilation (Panel **(C)**) in freely-moving rats prior to (pre) and following injection of morphine (10 mg/kg, IV) and subsequent injections of vehicle (saline, IV) or D-cysteine ethyl ester (D-CYSee, 500 μmol/kg, IV). Data are presented as mean ± SEM. There were 5 rats in the vehicle group and 6 rats in the D-CYSee group.

The baseline (pre) ventilatory parameters before injecting vehicle or D-CYSee (500 μmol/kg, IV) are shown in Supplementary Table S2. There were no between group differences for any parameter (*p* > 0.05, for all comparisons *via* one-way ANOVA). The responses elicited by morphine in the 15-min period before giving vehicle or D-CYSee are shown in Supplementary Tables S3, S4. Descriptions of individual responses to morphine are given below. Moment-to-moment values of frequency of breathing (Freq), tidal volume (TV), and minute ventilation (MV) before and after injection of morphine (10 mg/kg, IV) and then vehicle or D-CYSee are shown in Figure 1. Morphine elicited a transient increase in Freq followed by a sustained decrease that was present when vehicle or D-CYSee was given (Figure 1A). The vehicle injection did not elicit a response in Freq, which recovered to pre-injection values within 20–25 min. D-CYSee elicited brief decreases in Freq for about 1 min followed by a sustained rise in Freq to pre-morphine values. Morphine elicited a sustained fall in TV that was pronounced at injection of vehicle or D-CYSee (Figure 1B). The injection of vehicle did not immediately change TV, which gradually recovered to pre-injection levels within 90 min. The first injection of D-CYSee elicited a prompt and sustained reversal of the effects of morphine on TV, and the second injection elicited a minor further increase. TV values stayed at or above baseline over the post-D-CYSee injection phases. As a result of the changes in Freq and TV, morphine elicited a sustained decrease in MV in vehicle-treated rats, whereas D-CYSee elicited a prompt and sustained reversal of the adverse effects of morphine on MV (Figure 1C).

**FIGURE 2:**
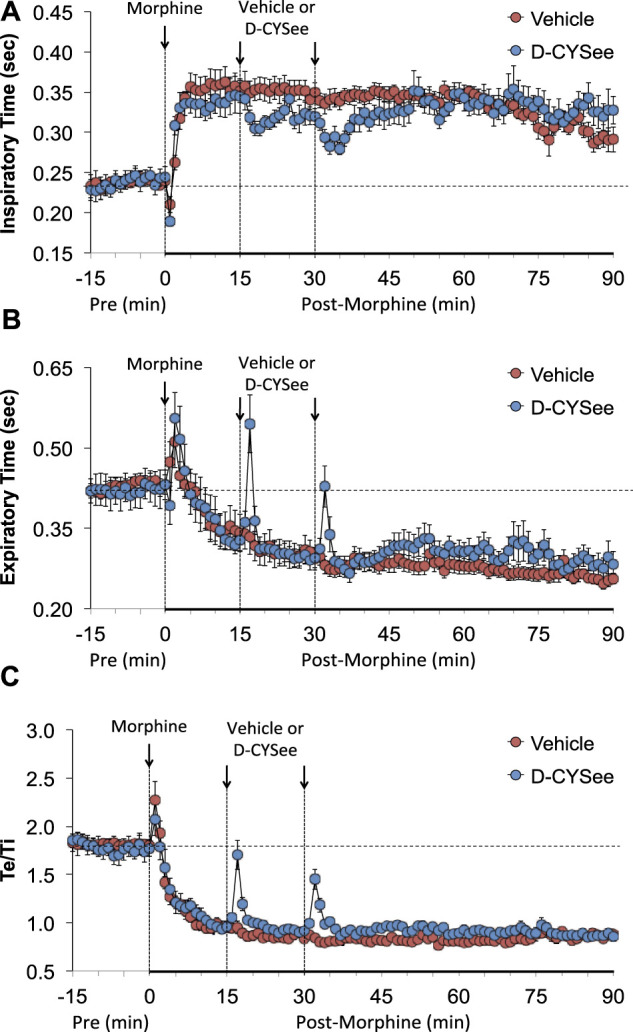
Values of inspiratory time (Panel **(A)**), expiratory time (Panel **(B)**), and expiratory time/inspiratory time (Te/Ti) (Panel **(C)**) in freely-moving rats prior to (pre) and following injection of morphine (10 mg/kg, IV) and subsequent injections of vehicle (saline, IV) or D-cysteine ethyl ester (D-CYSee, 500 μmol/kg, IV). Data are presented as mean ± SEM. There were 5 rats in the vehicle group and 6 rats in the D-CYSee group.

As shown in Figure 2A, morphine elicited a transient decrease in Ti followed by sustained increases in rats that received vehicle 15 min after injection of morphine. The first and second injections of D-CYSee elicited transient decreases in Ti. The long-lasting rise in Ti elicited by morphine between 45 and 90 min post-injection was essentially similar in vehicle- and D-CYSee–treated rats. As shown in Figure 2B, morphine elicited a transient increase in Te followed by a long-lasting decrease in Te in rats that received vehicle 15 min after injection of morphine. The injections of D-CYSee elicited brief increases in Te, but minimally affected the long-lasting morphine-induced decrease in Te. As shown in Figure 2C, the respiratory quotient (Te/Ti) fell markedly after injection of morphine in vehicle-treated rats and similarly in D-CYSee-treated rats, while it was evident that the injections of D-CYSee elicited brief increases in Te/Ti. As shown in Supplementary Figure S2A, morphine elicited a sustained increase in end inspiratory pause (EIP). The injections of D-CYSee elicited transient decreases in EIP. As seen in Supplementary Figure S2B, morphine elicited robust transient, increases in end expiratory pause (EEP) followed by sustained decreases in EEP. The two injections of D-CYSee elicited minor initial increases in EEP, but did not alter the long-term decrease in EEP.

**FIGURE 3:**
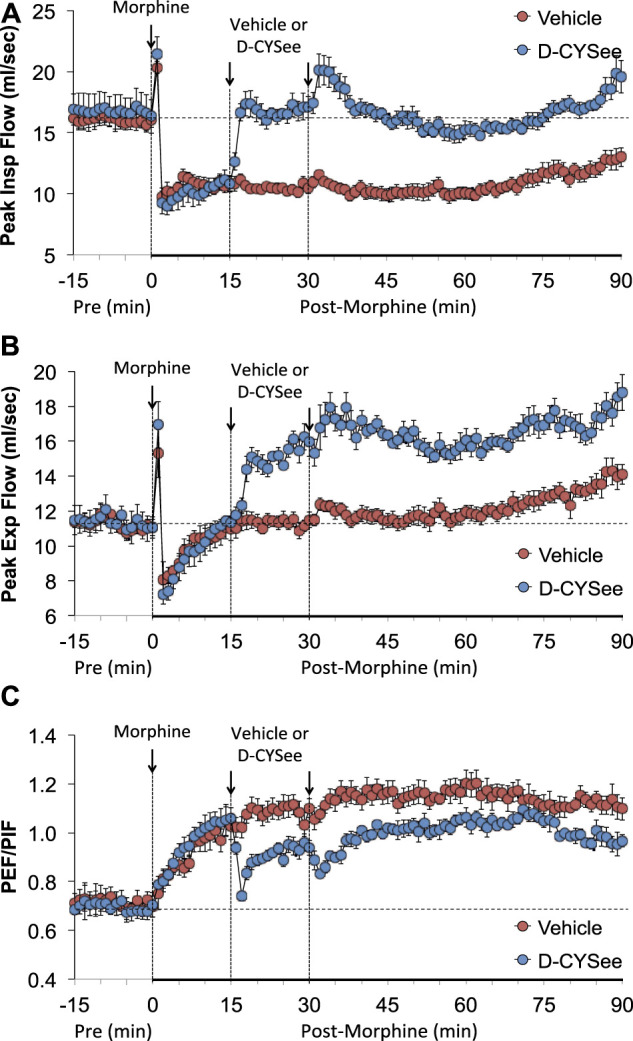
Values of peak inspiratory flow (Panel **(A)**), peak expiratory flow (Panel **(B)**), and peak expiratory flow/peak inspiratory flow (PEF/PIF) (Panel **(C)**) in freely-moving rats prior to (pre) and following injection of morphine (10 mg/kg, IV) and subsequent injections of vehicle (saline, IV) or D-cysteine ethyl ester (D-CYSee, 2 × 500 μmol/kg, IV). The data are presented as mean ± SEM. There were 5 rats in the vehicle group and 6 rats in the D-CYSee group.

**FIGURE 4:**
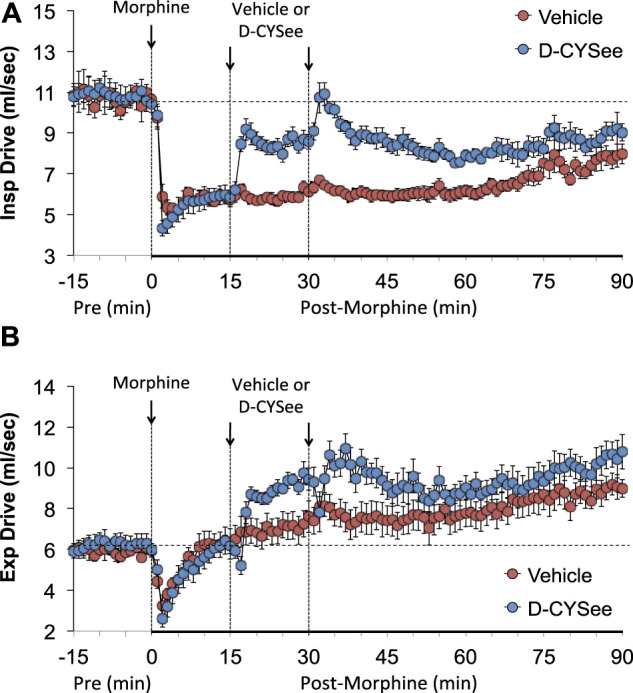
Calculated values of inspiratory drive (TV/Ti) (Panel **(A)**) and expiratory drive (TV/Te) (Panel **(B)**) in freely moving rats prior to (pre) and following injection of morphine (10 mg/kg, IV) and subsequent injections of vehicle (saline) or D-cysteine ethyl ester (D-CYSee, 500 μmol/kg, IV). The data are presented as mean ± SEM. There were 5 rats in the vehicle group and 6 rats in the D-CYSee group.

As seen in Figure 3, morphine elicited a pronounced and sustained fall in PIF (Figure 3A) but a transient decrease in PEF (Figure 3B) in vehicle-treated rats. The injections of D-CYSee elicited a prompt and sustained reversal of the effects of morphine on PIF and a marked increase in PEF to levels above pre-morphine values. Morphine elicited a prompt and sustained increase in PEF/PIF ratios (Figure 3C) in vehicle-treated rats. D-CYSee elicited robust decreases in PEF/PIF that were not sustained throughout the entire recording period. As shown in Figure 4, morphine elicited a prompt and sustained decrease in inspiratory drive (Figure 4A) and a pronounced transient decreases in expiratory drive (Figure 4B). D-CYSee caused partial recovery of inspiratory drive, and a sustained increase in expiratory drive to above pre-morphine levels. As shown in Supplementary Figure S3A, morphine elicited a sustained increase in EF_50_ that remained elevated in rats that received vehicle. The first injection of D-CYSee elicited a prompt and sustained increase in EF_50_ that was unchanged with the second injection. As seen in Supplementary Figure S3B, morphine elicited a prompt and sustained decrease in relaxation time. Vehicle injections elicited minimal responses, whereas D-CYSee elicited transient increases in relaxation time. As seen in Supplementary Figure S3C, morphine elicited a prompt and, after a noticeable decline in peak response, sustained increase in apneic pause. As seen in Supplementary Figure S3D, morphine elicited a prompt and transient increase in expiratory delay. The injections of vehicle elicited minimal changes in both apneic pause and expiratory delay, whereas D-CYSee elicited transient increases in both parameters. As seen in Supplementary Figure S4, morphine elicited robust increases in NEBI (Supplementary Figure S4A) and NEBI/Freq (Supplementary Figure S4B) of about 5 min in duration followed by sustained decreases in these parameters. The injections of vehicle or D-CYSee elicited minor changes in these parameters.

**FIGURE 5:**
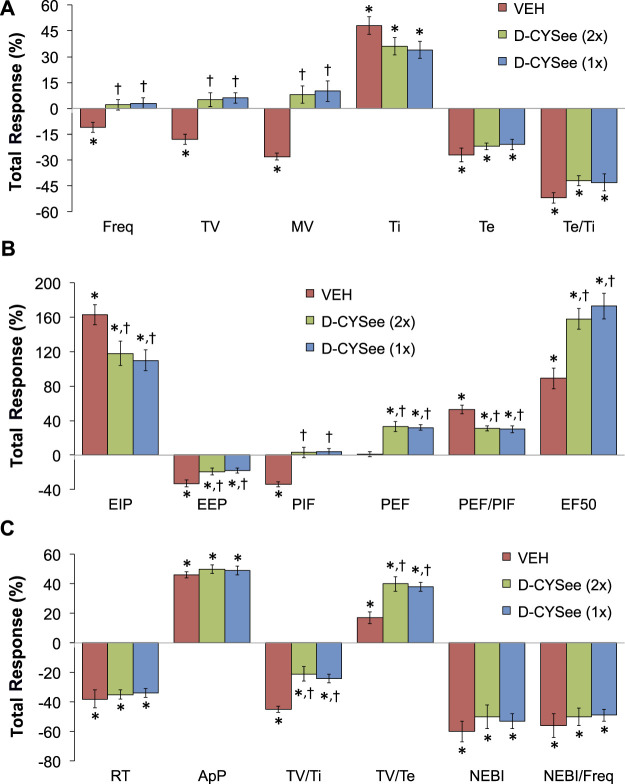
Total changes (expressed as % change from pre-values) in Freq, TV, MV, Ti, Te and Te/Ti **(A)**, EIP, EEP, PIF, PEF, PEF/PIF and EF50 **(B)**, and RT, ApP, TV/Ti, TV/Te, NEBI, and NEBI/Freq **(C)** that occurred over the 15-min period following the first injection of vehicle (VEH, IV) or D-cysteine ethyl ester (D-CYSee, 500 μmol/kg, IV). The vehicle (VEH) group received two injections of vehicle. The D-CYSee (2x) group received two injections of D-CYSee, whereas the D-CYSee (1x) group received one injection of D-CYSee and then an injection of vehicle. There were 5 rats in the vehicle group and 6 rats in the D-CYSee groups. *p < 0.05, significant change from pre. ^†^ *p* < 0.05, D-CYSee versus vehicle.

**FIGURE 6:**
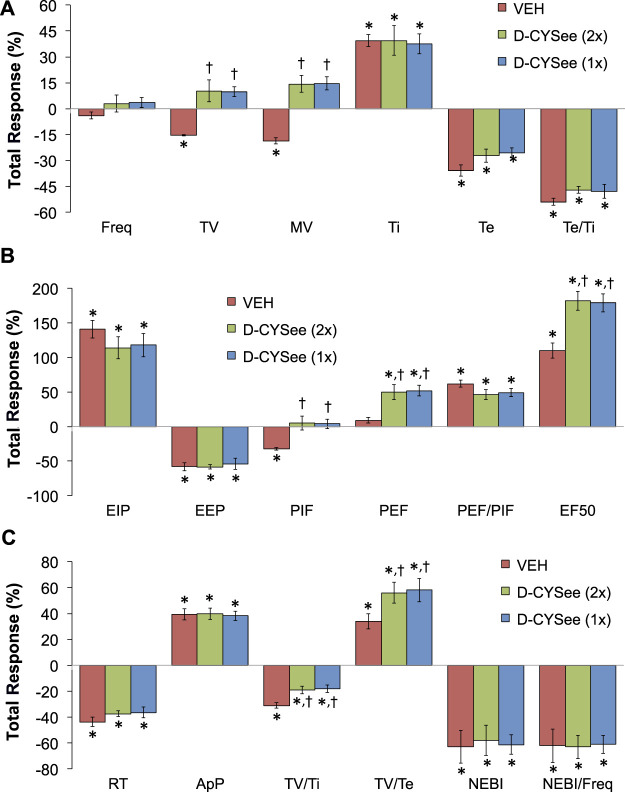
Total changes (expressed as % change from pre-values) in ventilatory parameters that occurred over the 60-min period following the second set of injections of either vehicle (VEH) or D-cysteine ethyl ester (D-CYSee, 500 μmol/kg, IV). The vehicle (VEH) group received two injections of vehicle. The D-CYSee (2x) group received two injections of D-CYSee, whereas the D-CYSee (1x) group received one injection of D-CYSee and then an injection of vehicle. There were 5 rats in the vehicle group and 6 rats in the D-CYSee group. *p < 0.05, significant change from pre-values. ^†^ *p* < 0.05, D-CYSee versus vehicle.

The total changes in ventilatory parameters recorded over the 15-min period following the first injection of vehicle or D-CYSee (expressed as % change from pre-values) are summarized in Figure 5. The three groups are those that received two injections of vehicle (VEH group), two injections of D-CYSee (D-CYSee (2x) group), and one injection of D-CYSee (D-CYSee (1x) group). As seen in Figure 5A, the morphine-induced decreases in Freq, TV and MV were significantly diminished in D-CYSee (2x) and D-CYSee (1x) groups, whereas changes in Ti, Te and Te/Ti were only marginally diminished (combined alterations in Ti and Te in D-CYSee (2x) and D-CYSee (1x) groups were significant and consistent with reversal of the decrease in Freq in each group, *p* < 0.05 for both comparisons via two-way ANOVA). As seen in Figure 5B, the morphine-induced changes in EIP, EEP, PIF, PEF, PEF/PIF and EF_50_ were all substantially reversed in D-CYSee (2x) and D-CYSee (1x) groups. As seen in Figure 5C, the morphine-induced decreases in inspiratory drive (TV/Ti) were diminished in D-CYSee (2x) and D-CYSee (1x) groups, and morphine-induced increases in expiratory drive (TV/Te) were augmented in D-CYSee (2x) and D-CYSee (1x) rats. In contrast, total changes in relaxation time (RT), apneic pause (ApP), NEBI, and NEBI/Freq were similar in the three groups. The total changes in ventilatory parameters recorded over the 60-min period after the second set of injections (% change from pre-values) are summarized in Figure 6. As seen in Figure 6A, the morphine-induced decreases in TV and MV were diminished in the D-CYSee (2x) and D-CYSee (1x) groups. There were minimal changes in Freq, and the pronounced increases in Ti and decreases in Te were similar in all three groups. As seen in Figure 6B, the decreases in PIF were smaller in D-CYSee (2x) and D-CYSee (1x) rats, whereas the increases in PEF and EF_50_ were augmented in D-CYSee (2x) and D-CYSee (1x) rats. The morphine-induced changes in EIP, EEP, and PEF/PIF were similar in all three groups. The resting values for Freq, TV, and MV prior to injection of vehicle or D-cysteine (500 umol/kg, IV) are presented in the legend of Supplementary Figure S5. There were no between group differences in any parameter (*p* > 0.05, for all comparisons via one-way ANOVA). As seen in Supplementary Figure S5, morphine (10 mg/kg, IV) elicited substantial falls in Freq (Supplementary Figure S5A), TV (Supplementary Figure S5B), and MV (Supplementary Figure S5C) that were not affected by the injections of D-cysteine.

### Effects of D-CYSee and D-Cysteine on Morphine-Induced Changes in ABG Chemistry and A-a Gradient

**FIGURE 7:**
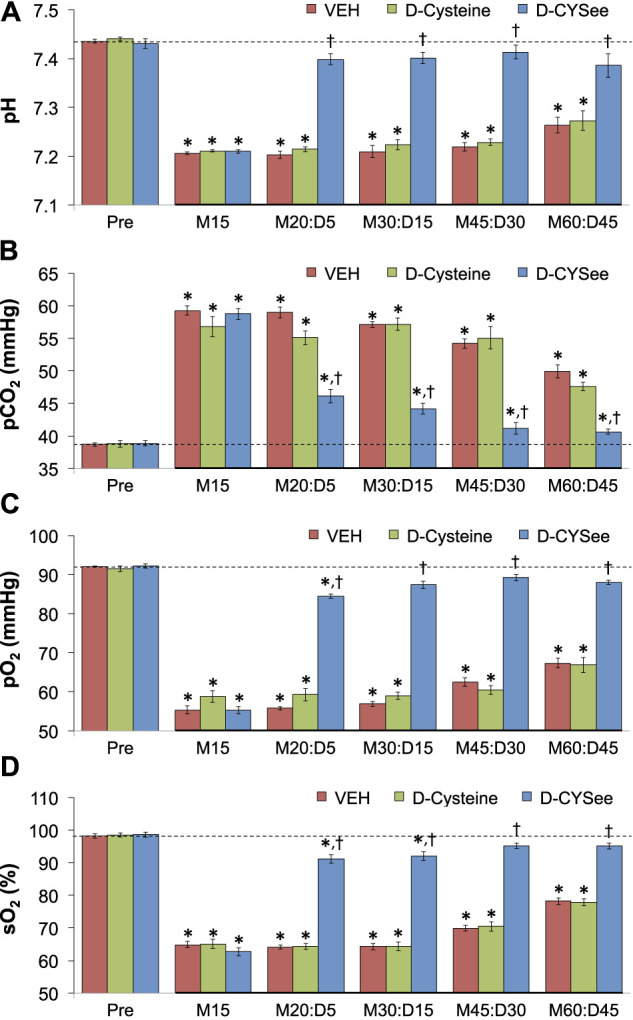
Values of pH (Panel **(A)**), pCO_2_ (Panel **(B)**), pO_2_ (Panel **(C)**), and sO_2_ (Panel **(D)**) before (Pre) and after injection of morphine (10 mg/kg, IV) in three separate groups of freely-moving rats followed by injection of vehicle (VEH (saline), IV), D-cysteine (500 μmol/kg, IV), or D-cysteine ethyl ester (D-CYSee, 500 μmol/kg, IV). The terms M15, M20, M30, M45, and M60 denote 15, 30, 45, and 60 min after injection of morphine. The terms D5, D15, D30, and D45 denote 5, 15, 30, and 45 min after injection of the drug (vehicle, D-cysteine, or D-CYSee). The data are mean ± SEM. There were 8 rats in the vehicle- or D-CYSee–treated groups and 6 rats in the D-cysteine–treated group. *p < 0.05, significant change from pre-values. ^†^ *p* < 0.05, D-CYSee versus vehicle.

**FIGURE 8:**
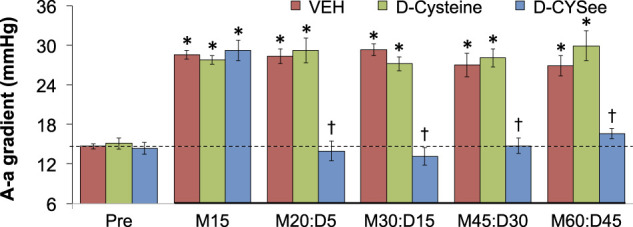
Alveolar–arterial (A-a) gradient values before (Pre) and after injection of morphine (10 mg/kg, IV) in three groups of freely-moving rats followed by an injection of vehicle (VEH (saline), IV), D-cysteine (500 μmol/kg, IV), or D-cysteine ethyl ester (D-CYSee, 500 μmol/kg, IV). The terms M15, M20, M30, M45, and M60 denote 15, 30, 45, and 60 min after injection of morphine. The terms D5, D15, D30, and D45 denote 5, 15, 30, and 45 min after injection of drug (vehicle, D-cysteine, or D-CYSee). The data are mean ± SEM. There were 8 rats in the vehicle- or D-CYSee-treated groups and 6 rats in the D-cysteine–treated group. *p < 0.05, significant change from pre-values. ^†^ *p* < 0.05, D-CYSee versus vehicle.

ABG values (pH, pCO_2_, pO_2_ and sO_2_) in three groups of freely-moving rats prior to and after injection of morphine (10 mg/kg, IV) and subsequent injection of vehicle (VEH, IV), D-cysteine (500 μmol/kg, IV), or D-CYSee (500 μmol/kg, IV) are summarized in Figure 7. The values denoted M15–M60 are the times post-morphine, whereas values denoted D5–D45 are the times post-vehicle/drug injection. As seen in Figure 7A, morphine elicited a substantial fall in pH in the three groups, and D-CYSee elicited a prompt and sustained reversal of the acidosis. As seen in Figure 7B, morphine elicited a substantial increase in pCO_2_ in the three groups and D-CYSee elicited an immediate and sustained reversal of the hypercapnia. As seen in Figures 7C,D, morphine elicited sustained decreases in pO_2_ and sO_2_, respectively, and D-CYSee elicited a prompt and sustained reversal of this hypoxemia. In contrast, the time-dependent trends toward pre-morphine values for pH, pCO_2_, pO_2_, and sO_2_ were similar in rats that received vehicle or D-cysteine. The A-a gradient values (index of alveolar gas exchange) in three groups of freely-moving rats before and after injection of morphine (10 mg/kg, IV) and then injections of vehicle (VEH, IV), D-cysteine (500 μmol/kg, IV), or D-CYSee (500 μmol/kg, IV) are shown in Figure 8. Morphine caused a sustained increase in A-a gradient, and D-CYSee elicited an immediate and sustained reversal of this effect, whereas vehicle and D-cysteine did not.

### Effects of D-CYSee and D-Cysteine on Antinociceptive Responses to Morphine

**FIGURE 9:**
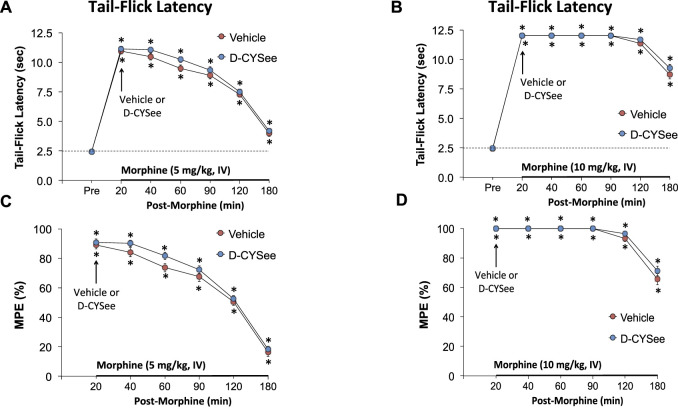
Tail-flick latencies elicited by intravenous injection of 5 mg/kg (Panel **(A)**) or 10 mg/kg (Panel **(B)**) of morphine in freely-moving rats that received injections of vehicle (VEH (saline), IV) or D-CYSee (500 μmol/kg, IV) 20 min after the injection of morphine. Panels **(C,D)** display the data as percentage maximum possible effect (%MPE). The data are shown as mean ± SEM. There were 6 rats in each group. *p < 0.05, significant change from pre-values. There were no between group comparisons at any time-point (*p* > 0.05, for all comparisons).

The temporal effects of a 5- or 10-mg/kg dose of morphine on TFL and subsequent effects elicited by an injection of vehicle or D-CYSee (500 μmol/kg, IV) are summarized in Figure 9. The 5-mg/kg (Figures 9A,C) and 10-mg/kg (Figures 9B,D) doses of morphine both elicited pronounced antinociception with the effects of the 10-mg/kg dose being more pronounced than the 5-mg/kg dose. As can be seen, the temporal changes in TFL elicited by either dose of morphine were similar in rats that received vehicle or D-CYSee.

### Effects of D-CYSee and D-Cysteine on the Sedative Effects of Morphine

The general behaviors of the rats that received morphine plus vehicle, D-cysteine or D-CYSee were similar to one another. Morphine caused an immediate (within 2 min) sedative effect in all rats that was manifested as a lack of mobility and unusual body postures. The full return of the righting reflex in vehicle-treated rats (72.3 ± 9.2 min, *n* = 12), D-cysteine (500 μmol/kg, IV)–treated rats (70.5 ± 10.2 min, *n* = 12), and D-CYSee (500 μmol/kg, IV)–treated rats (85.6 ± 8.6 min, *n* = 12) was equal to one another (*p* > 0.05, for all between-group comparisons via two-way ANOVA).

## Discussion

### General Observations About D-CYSee and Thiol esters

Our study provides evidence that D-CYSee produces a rapid and long-lasting reversal of the deleterious changes in breathing, ABG chemistry, and alveolar gas exchange (i.e., a reversal of the elevated A-a gradient) elicited by a 10-mg/kg dose of morphine in freely moving (unanesthetized) male Sprague–Dawley rats. However, D-CYSee did not greatly alter the sedative antinociceptive or effects of this dose of morphine (the antinociceptive effects were slightly augmented). Since the above actions of morphine are immediately reversed by systemic injection of OR antagonists (van Dorp et al., 2007; Dahan et al., 2010, 2018; Boom et al., 2012; Algera et al., 2019), it is unlikely that D-CYSee alters the actions of morphine by competitive or non-competitive antagonism of ORs. We have no evidence as to the physiological site(s) of action or molecular processes by which D-CYSee profoundly reverses morphine-induced changes in breathing, ABG chemistry, and A-a gradient, while slightly augmenting the antinociceptive effects of the 10-mg/kg dose of this opioid. There is evidence that morphine-induced inhibition of excitatory amino acid transporter 3 (Trivedi et al., 2014; Trivedi and Deth, 2015) blocks L-cysteine entry into cells, which suggests that the adverse effects of morphine involve (1) reduced intracellular L-cysteine levels and accompanying increase in oxidative status of the cell (Trivedi et al., 2014; Trivedi and Deth, 2015), and/or (2) diminished involvement of L-cysteine in metabolic pathways in cells, such as generation of hydrogen sulfide (Wu, 2009; Yin et al., 2016; Paul et al., 2018). Since the antinociceptive effects of a 5-mg/kg dose of morphine were slightly shorter in duration in D-CYSee-treated rats than in vehicle-treated rats, it suggests that the 10-mg/kg dose of morphine may have masked some adverse effects of D-CYSee with respect to antinociceptive signaling.

### Potential Mechanisms of Action of D-CYSee and Other Thiol esters

Although the abilities of D-CYSee and L-CYSee (Mendoza et al., 2013) to reverse the deleterious actions of morphine on breathing and alveolar gas exchange could involve supplying reducing equivalents to target cells, we found that the potent reducing and cell-permeable thiol ester, N-acetyl-L-cysteine methyl ester (L-NACme) (Tsikas et al., 2018), had minimal effects on the ventilatory-depressant effects of morphine (Gaston et al., 2021). The readily conversion of L-NACme to L-cysteine in cells (Lailey and Upshall, 1994) argues that the effects of D-CYSee are not due to conversion to H2S via the D-amino acid oxidase–mercaptopyruvate sulfur transferase system (Shibuya and Kimura, 2013; Kimura, 2014). Since the superoxide anion/free radical scavenger, Tempol, attenuates OIRD elicited by morphine and fentanyl (Baby et al., 2021a,b), it points to the importance of redox mechanisms in the actions of opioids and potentially the ability of thiol esters to reverse OIRD. Aside from direct interactions with functional proteins, potential mechanisms of action of D-CYSee may involve (1) direct binding to myristoylated alanine-rich C-kinase substrate (MARCKS), which is a D-cysteine binding protein (Semenza et al., 2021); (2) modulation of OR-β-arrestin cell signaling events that spare the analgesic G-protein–mediated actions of morphine (Schmid et al., 2017; Grim et al., 2020); and/or (3) conversion of D-CYSee to S-nitroso-D-CYSee by nitric oxide synthase-dependent processes (Perissinotti et al., 2005; Hess and Stamler, 2012; Stomberski et al., 2019; Seckler et al., 2021), which may act similar to the intracellular S-nitrosylating agent, S-nitroso-L-CYSee (Clancy et al., 2001). We determined whether D-CYSee increases NADPH diaphorase expression in tissues, which identifies S-nitrosylated species (both free S-nitrosothiols and S-nitrosylated proteins) in aldehyde-treated tissues (Seckler and Lewis, 2020). S-nitrosothiols, such as S-nitroso-L-cysteine and S-nitroso-L-glutathione, play key roles in ventilatory control processes in brainstem and peripheral structures, such as the carotid bodies (Palmer et al., 2013; Gaston et al., 2014; Palmer et al., 2015; Gaston et al., 2020; Gaston et al., 2021). We reported that the ability of morphine to depress breathing and adversely affect ABG chemistry is reduced in rats undergoing intravenous infusion of S-nitroso-L-cysteine (Getsy et al., 2022), and that whereas S-L-nitrosothiols exert pronounced effects on cardiorespiratory systems, D-isomers exert minimal effects (Davisson et al., 1996; Lewis et al., 1996; Travis et al., 1996; Davisson et al., 1997; Ohta et al., 1997; Travis et al., 1997; Lewis et al., 2005; Lewis et al., 2006; Gaston et al., 2020; Gaston et al., 2021).

Our findings that D-cysteine did not mimic the effects of D-CYSee suggest that (1) D-CYSee activates membrane-bound functional proteins not activated by D-cysteine; (2) D-CYSee acts by entering cells and modulating intracellular signaling pathways as the thiol ester; (3) D-cysteine is unable to enter into cells via uptake systems available to L-cysteine (Tunnicliff, 1994; Shanker and Aschner, 2001; Aoyama et al., 2008; Albrecht and Zielińsk, 2019), including high-affinity sodium-dependent glutamate transporters (e.g., GLT1 and GLAST) (Hargreaves et al., 1988), excitatory amino acid transporters 1–3 (Zerangue and Kavanaugh, 1996; Chen and Swanson, 2003; Himi et al., 2003; Trivedi et al., 2014; Trivedi and Deth, 2015; Albrecht and Zielińsk, 2019), large neutral amino acid transporters, LAT1 and LAT2 (Simmons-Willis et al., 2002; Nemoto et al., 2003; Li and Whorton, 2007; Granillo et al., 2008), and the band 3 protein/anion transport system (Young et al., 1981; Tunnicliff, 1994), with the only direct evidence being that D-cysteine is not transported by LAT1 or LAT2 (Simmons-Willis et al., 2002); and (4) D-cysteine cannot modulate the activity of intracellular proteins modulated by D-CYSee. We are currently exploring whether the formation of D-CYSee: D-glucose and/or D-cysteine: D-glucose, similar to L-cysteine: D-glucose (Gomez et al., 1994; Wróbel et al., 1997; Szwergold, 2006; Li et al., 2015) contributes to the effects of D-CYSee.

### D-CYSee Reverses the Deleterious Effects of Morphine on Ventilatory Parameters

As expected (May et al., 2013a,b; Young et al., 2013; Baby et al., 2018, Baby et al., 2021 SM.; Gaston et al., 2021), a 10-mg/kg dose of morphine elicited a relatively transient decrease in Freq, which was not truly indicative of the actual effects of morphine, since morphine elicited sustained increases in Ti (lengthened inspiration), whereas it elicited pronounced decreases in Te (shortened expiration). Morphine also elicited a sustained increase in EIP, but a relatively transient decrease in EEP followed by a sustained decrease. The propensity of morphine to elongate Ti while not greatly affecting Te (Fone and Wilson, 1986; Kasaba et al., 1997; Chevillard et al., 2009) or shortening Te (May et al., 2013a,b; Young et al., 2013; Baby et al., 2018, Baby et al., 2021 SM.; Gaston et al., 2021) is known, as is the ability of opioids to differentially affect EIP and EEP (Henderson et al., 2013, 2014; May et al., 2013a,b; Young et al., 2013; Baby et al., 2018, Baby et al., 2021 SM.; Gaston et al., 2021). The molecular mechanisms and brain sites, such as the nucleus tractus solitarius (Hassan et al., 1982; Li et al., 1996; Iniushkin, 1997; Zhuang et al., 2012), pre-Bötzinger complex (Bachmutsky et al., 2020; Varga et al., 2020), and parabrachial nucleus/Kölliker–Fuse nucleus (Eguchi et al., 1987; Lalley et al., 2014; Bachmutsky et al., 2020; Varga et al., 2020), by which the differential effects of opioids (e.g., morphine and fentanyl) on inspiratory and expiratory timing are accomplished have received attention, and the conclusion is that the qualitative/quantitative effects of opioids on Ti and Te are dose-dependent (Li et al., 1996; Henderson et al., 2013, 2014). In contrast, the sites and mechanisms by which opioids differentially affect EIP and EEP have received limited attention (Henderson et al., 2014; Bachmutsky et al., 2020). Although studies in anesthetized rats and in *in vitro* preparations suggest that the ability of morphine to decrease Freq involves depression of carotid body chemoreceptor afferent reflexes, the ability of morphine (10 mg/kg, IV) to depress breathing in unanesthetized rats is enhanced in rats, which had undergone prior bilateral carotid sinus nerve transection (Baby et al., 2018), suggesting that morphine promotes rather than inhibits carotid body chemoreflex activity in this circumstance.

The findings that the injections of D-CYSee elicited transient decreases in Freq in morphine-treated rats, and that both responses were followed by sustained reversal of the depressant effects of morphine, points to D-CYSee having multiple mechanisms of action that are not determined as of yet. The brief decreases in Freq were associated primarily with brief increases in Te, relaxation time, apneic pause, and expiratory delay, whereas Ti and EIP fell at these times, and EEP changed minimally. It appears that D-CYSee initially targets mechanisms that regulate some aspects of expiratory timing in morphine-treated rats, and it does not adversely impact inspiratory timing control processes. In contrast, the ability of D-CYSee to reverse the adverse effects of morphine on Freq was almost totally due to the reversal of the enhanced Ti, as D-CYSee elicited relatively minor effects on Te, EIP, EEP, relaxation time, apneic pause or expiratory delay. As such, it would be of great interest to determine whether microinjections of D-CYSee into key brainstem nuclei, such as the nucleus tractus solitarius, pre-Bötzinger complex, Kölliker–Fuse nucleus, and/or parabrachial nucleus, are able to modulate the effects of morphine on respiratory timing and other parameters.

As reported, morphine elicited sustained decreases in TV, MV and PIF; sustained increases in EF_50_; and transient decreases in PEF (May et al., 2013a,b; Young et al., 2013; Baby et al., 2018, Baby et al., 2021 SM.; Gaston et al., 2021). This pattern of responses speaks to the disparate role of OR signaling mechanisms in the control of breathing. Another key set of findings pertains to the first injection of D-CYSee eliciting a rapid and sustained reversal of the adverse effects of morphine on TV, MV and PIF, and augmentation of PEF and EF_50_ to values that were above those of morphine alone, with a second injection of D-CYSee generally maintaining the response. We conclude that the second injection of D-CYSee was not necessary for full effect as shown by responses in rats that received a single dose of D-CYSee. Our unpublished data shows that the first injections of a 500-μmol/kg dose of L-cysteine ethyl ester or L-cysteine methyl ester elicit similar responses to D-CYSee, whereas the second injection of the L-thiol esters elicits pronounced ventilatory responses in morphine-treated rats. This suggests that the second dose of L-isomers activates signal/enzymatic pathways that D-CYSee cannot. We also found that morphine elicited a pronounced, but transient increase in NEBI, likely due to greater occurrence of apneas (Henderson et al., 2013, 2014; Gaston et al., 2021) followed by a sustained decrease in this index of respiratory instability. Thus, D-CYSee had no effects on morphine promotion of eupneic breathing.

### D-CYSee Reverses the Deleterious Effects of Morphine on ABG Chemistry and A-a Gradient

As expected from our previous studies in rats (Gaston et al., 2021; Getsy et al., 2022), the bolus intravenous injection of morphine elicited pronounced and long-lasting deleterious changes in ABG chemistry (i.e., a decrease in pH, an increase in pCO_2_, and decreases in pO_2_ and sO_2_) that are consistent with hypoventilation, and accompanied by a sustained increase in A-a gradient, which indicates a problem with alveolar gas exchange (Gaston et al., 2021; Getsy et al., 2022). This increase in A-a gradient could be due to alveolar collapse (atelectasis) because of hypoventilation (Gaston et al., 2021; Getsy et al., 2022) or may also involve more complicated effects on surfactant status and/or alveolar fluid clearance, which if reduced would impair gas exchange in alveoli that remain open, despite a decreased TV. As such, the second set of key findings was that D-CYSee elicited an immediate and sustained reversal of the adverse effects of morphine on ABG chemistry and A-a gradient. These findings are important since improved ABG chemistry is the final arbiter of how well a reversal agent combats OIRD. Whether the improved (normalized) status of ABG chemistry and A-a gradient is due to improved ventilation and reversal of atelectasis or whether D-CYSee directly affects alveoli signaling processes controlling surfactant and fluid status is unknown.

### D-CYSee Does Not Affect the Antinociceptive Actions of Morphine

The third set of key findings pertains to the lack of effect of D-CYSee on morphine antinociception. As expected, the injections of the 5- or 10-mg/kg doses of morphine elicited robust increases in TFL (antinociceptive effects) of over 180 min in duration (Gaston et al., 2021). D-CYSee (500 μmol/kg, IV) did not alter the temporal changes in TFL elicited by 5- or 10-mg/kg doses of morphine. There is no information about how D-CYSee affects nociceptive signaling although Pathirathna et al. (2006) found that intra-dermal injections of D-cysteine into the ventral side of a hind paw (peripheral receptive field) of rats caused dose-/time-dependent hyperalgesia that was blocked by 3-betaOH, a neuroactive steroid that blocks voltage-dependent T-type Ca^2+^ channels. These and other data (Todorovic and Jevtovic-Todorovic, 2014) imply that changes in redox status of nociceptors may function as a local intrinsic mechanism in controlling peripheral pain perception. Our data suggest that D-CYSee does not cause (1) dynamic changes in the functional status of ORs (e.g., phosphorylation, desensitization, and internalization events), and (2) modulation of intracellular signaling cascades by which morphine elicits its antinociception effects (Connor and Christie, 1999; Williams et al., 2013).

## Study Limitations

Our findings that a 500-μmol/kg dose of D-CYSee reverses the adverse actions of morphine on breathing, ABG chemistry and alveolar gas exchange need to be evaluated at lower doses. The ability of D-CYSee to reverse the adverse effects of higher potency opioids, such as fentanyl and carfentanil, should be determined, especially considering the key role that these synthetic opioids are playing in the current opioid epidemic (Arendt, 2021; Deo et al., 2021). Another important limitation is the lack of evidence as to the efficacy of D-CYSee in reversing OIRD elicited by morphine and fentanyl in female rats knowing that opioids can exert qualitatively/quantitatively different responses in females than males (Dahan et al., 1998; Hosseini et al., 2011). Another limitation pertains to our lack of understanding of molecular mechanisms by which D-CYSee modulates the actions of opioids. We are examining the extent to which systemic D-CYSee generates S-nitrosothiols in brain structures such as, the nucleus tractus solitarius, and peripheral structures such as, the carotid bodies, by NADPH diaphorase histochemistry (Seckler et al., 2020) and sensor technology (Seckler et al., 2017). At present, we do not know whether the ability of D-CYSee to reverse morphine-induced respiratory depression is due to its direct respiratory stimulant effects and/or its ability to counteract the inhibitory actions of µ-opioid receptor activation. We have preliminary data that the injection of D-CYSee (500 μmol/kg, IV) elicits a prompt increase in breathing in freely moving rats that peaks at 2 min (+59.6 ± 7.3%, *n* = 6 rats, *p* < 0.05), but subsides before 10 min and at 15 min post-injection, the values were +3.6 ± 7.3% of pre-injection values (*p* > 0.05). However, despite the recovery to baseline, the injection of morphine at the 15-min post-D-CYSee time point elicited markedly smaller decreases in MV than in vehicle-injected rats. There was a maximal decrease in MV in the vehicle- and D-CYSee-injected rats being −61.5 ± 7.3% (*p* < 0.05) and −23.8 ± 5.4%, *p* < 0.05, respectively (*p* < 0.05, comparing D-CYSee vs. vehicle). Moreover, the return to baseline levels after the injection of morphine occurred within 73.7 ± 9.4 min in vehicle-treated rats, but only 12.3 ± 3.6 min in D-CYSee-treated rats (*p* < 0.05, comparing D-CYSee vs. vehicle). Clearly, this means that despite the recovery of MV to baseline following the administration of D-CYSee, the D-thiol ester or downstream products, such as S-nitroso-D-CYSee, may be present in sufficient quantities to countermand µ-opioid receptor activation. Ongoing pharmacokinetics studies using LC-MS methods available in our laboratory (Altawallbeh et al., 2019) are determining the temporal distribution of D-CYSee in the blood, peripheral organs and tissues, cerebrospinal fluid, and brain structures. Finally, we need to see whether D-CYSee can diminish the latent deleterious effects of morphine on ventilatory responses to hypoxic (May et al., 2013a) or hypoxic-hypercapnic (May et al., 2013b) gas challenges. Whether any pharmacological actions of D-CYSee involve conversion to the L-isomer is yet to be identified. As always, the most important concern is whether the pharmacological actions of OIRD reversal agents in rats will translate into effective therapies in humans. Many such OIRD reversal agents that have shown efficacy in rats did not show such efficacy in human clinical trials (Dahan et al., 2018; Algera et al., 2019).

## Conclusion

The systemic injection of D-CYSee reversed many of the adverse effects of morphine on ventilatory parameters, A-a gradient, and ABG chemistry in unanesthetized rats, whereas it did not markedly influence the antinociceptive or sedative actions of the opioid. Since D-cysteine did not affect the actions of morphine, it appears that as a readily cell-penetrant thiol ester, D-CYSee may modulate the adverse actions of morphine by interfering/reversing the downstream intracellular signaling processes by which morphine acts, rather than by directly blocking ORs. It is tempting to assume that D-CYSee reverses the adverse effects of morphine by entering the brain. However, since peripherally restricted OR antagonists, such as naloxone methiodide, blunt the cardiorespiratory and analgesic actions of opioids (Henderson et al., 2014), we suggest that the ability of D-CYSee to affect the cardiorespiratory actions of morphine involves interactions with peripheral OR signaling pathways such as those in the carotid bodies. These present findings with D-CYSee add to our knowledge about the efficacy of thiol esters (Gaston et al., 2021; Jenkins et al., 2021) and the superoxide anion/free-radical scavenger, Tempol (Baby et al., 2021a,b), against OIRD. Since D-CYSee did not reverse the antinociception or sedation elicited by morphine, it would appear that the ability of D-CYSee to reverse the deleterious effects of morphine on breathing is not due to direct antagonist actions at ORs. However, a key unresolved issue is whether D-CYSee reverses the deleterious effects of morphine on breathing via reversal of OR-mediated cell signaling events or simply by being a respiratory stimulant. We have unpublished data showing that the injection of D-CYSee increases MV in naïve rats, but that the effects subside within 10–15 min. Importantly, the subsequent injection of morphine elicited markedly smaller decreases in MV in these rats compared with naïve rats. Accordingly, it appears that D-CYSee can block morphine-induced respiratory depression independently of it being a respiratory stimulant *per se*. The mechanisms by which D-CYSee may interfere with OR-signaling events are under investigation.

## Data Availability

The raw data supporting the conclusion of this article will be made available by the authors, without undue reservation.

## Abbreviations

A-a gradient, alveolar–arterial gradient; ABG, arterial blood–gas chemistry; D-CYSee, D-cysteine ethyl ester; EEP, end-expiratory pause; EF_50_, expiratory flow at 50% expired tidal volume; EIP, end-inspiratory pause; FiO_2_, fraction of O_2_ in inspired air; Freq, frequency of breathing; HPL, hind-paw withdrawal latency; L-NACme, N-acetyl-L-cysteine methyl ester; MPE, maximal positive effect; MV, minute ventilation; NEBI, non-eupneic breathing index; OIRD, opioid-induced respiratory depression; P_atm_, atmospheric pressure; PEF, peak expiratory flow; P_H2O_, partial pressure of H_2_O in inspired air; PIF, peak inspiratory flow; RQ, respiratory quotient; RT, relaxation time; Te, expiratory time; TFL, tail-flick latency; Ti, inspiratory time; TV, tidal volume; and VEH, vehicle.

## References

[B1] Albrecht J. Zielińska M. (2019). Exchange-mode Glutamine Transport across CNS Cell Membranes. Neuropharmacology 161, 107560. 10.1016/j.neuropharm.2019.03.003 30853601

[B2] Algera M. H. Kamp J. van der Schrier R. van Velzen M. Niesters M. Aarts L. (2019). Opioid-induced Respiratory Depression in Humans: a Review of Pharmacokinetic-Pharmacodynamic Modelling of Reversal. Br. J. Anaesth. 122, e168–e179. 10.1016/j.bja.2018.12.023 30915997

[B3] Altawallbeh G. Smith L. Lewis S. J. Authier S. Bujold K. Gaston B. (2019). Pharmacokinetic Study of Sudaxine in Dog Plasma Using Novel LC-MS/MS Method. Drug Test. Anal. 11, 403–410. 10.1002/dta.2507 30242972

[B4] Aoyama K. Watabe M. Nakaki T. (2008). Regulation of Neuronal Glutathione Synthesis. J. Pharmacol. Sci. 108, 227–238. 10.1254/jphs.08r01cr 19008644

[B5] Arendt F. (2021). The Opioid-Overdose Crisis and Fentanyl: The Role of Online Information Seeking via Internet Search Engines. Health Commun. 36, 1148–1154. 10.1080/10410236.2020.1748820 32285691

[B6] Baby S. Gruber R. Discala J. Puskovic V. Jose N. Cheng F. (2021a). Systemic Administration of Tempol Attenuates the Cardiorespiratory Depressant Effects of Fentanyl. Front. Pharmacol. 12, 690407. 10.3389/fphar.2021.690407 34248639 PMC8260831

[B7] Baby S. M. Discala J. F. Gruber R. Getsy P. M. Cheng F. Damron D. S. (2021b). Tempol Reverses the Negative Effects of Morphine on Arterial Blood-Gas Chemistry and Tissue Oxygen Saturation in Freely-Moving Rats. Front. Pharmacol. 12, 749084. 10.3389/fphar.2021.749084 34630119 PMC8493249

[B8] Baby S. M. Gruber R. B. Young A. P. MacFarlane P. M. Teppema L. J. Lewis S. J. (2018). Bilateral Carotid Sinus Nerve Transection Exacerbates Morphine-Induced Respiratory Depression. Eur. J. Pharmacol. 834, 17–29. 10.1016/j.ejphar.2018.07.018 30012498 PMC6091892

[B9] Bachmutsky I. Wei X. P. Kish E. Yackle K. (2020). Opioids Depress Breathing through Two Small Brainstem Sites. Elife 9, e52694. 10.7554/eLife.52694 32073401 PMC7077984

[B10] Ben-Nun A. Bashan N. Potashnik R. Cohen-Luria R. Moran A. (1992). Cystine Dimethyl Ester Reduces the Forces Driving Sodium-dependent Transport in LLC-PK1 Cells. Am. J. Physiol. 263, C516–C520. 10.1152/ajpcell.1992.263.2.C516 1325121

[B11] Boom M. Niesters M. Sarton E. Aarts L. Smith T. W. Dahan A. (2012). Non-analgesic Effects of Opioids: Opioid-Induced Respiratory Depression. Curr. Pharm. Des. 18, 5994–6004. 10.2174/138161212803582469 22747535

[B12] Carstens E. Wilson C. (1993). Rat Tail Flick Reflex: Magnitude Measurement of Stimulus-Response Function, Suppression by Morphine and Habituation. J. Neurophysiol. 70, 630–639. 10.1152/jn.1993.70.2.630 8410163

[B13] Chapman C. D. Dono L. M. French M. C. Weinberg Z. Y. Schuette L. M. Currie P. J. (2012). Paraventricular Nucleus Anandamide Signaling Alters Eating and Substrate Oxidation. Neuroreport 23, 425–429. 10.1097/WNR.0b013e32835271d1 22395656

[B14] Chen Y. Swanson R. A. (2003). The Glutamate Transporters EAAT2 and EAAT3 Mediate Cysteine Uptake in Cortical Neuron Cultures. J. Neurochem. 84, 1332–1339. 10.1046/j.1471-4159.2003.01630.x 12614333

[B15] Chevillard L. Mégarbane B. Risède P. Baud F. J. (2009). Characteristics and Comparative Severity of Respiratory Response to Toxic Doses of Fentanyl, Methadone, Morphine, and Buprenorphine in Rats. Toxicol. Lett. 191, 327–340. 10.1016/j.toxlet.2009.09.017 19819313

[B16] Chu F. Chen L. H. O'Brian C. A. (2004). Cellular Protein Kinase C Isozyme Regulation by Exogenously Delivered Physiological Disulfides-Iimplications of Oxidative Protein Kinase C Regulation to Cancer Prevention. Carcinogenesis 25, 585–596. 10.1093/carcin/bgh041 14656938

[B17] Clancy R. Cederbaum A. I. Stoyanovsky D. A. (2001). Preparation and Properties of S-Nitroso-L-Cysteine Ethyl Ester, an Intracellular Nitrosating Agent. J. Med. Chem. 44, 2035–2038. 10.1021/jm000463f 11384248

[B18] Connor M. Christie M. D. (1999). Opioid Receptor Signalling Mechanisms. Clin. Exp. Pharmacol. Physiol. 26, 493–499. 10.1046/j.1440-1681.1999.03049.x 10405772

[B19] Dahan A. Aarts L. Smith T. W. (2010). Incidence, Reversal, and Prevention of Opioid-Induced Respiratory Depression. Anesthesiology 112, 226–238. 10.1097/ALN.0b013e3181c38c25 20010421

[B20] Dahan A. Sarton E. Teppema L. Olievier C. (1998). Sex-related Differences in the Influence of Morphine on Ventilatory Control in Humans. Anesthesiology 88, 903–913. 10.1097/00000542-199804000-00009 9579498

[B21] Dahan A. van der Schrier R. Smith T. Aarts L. van Velzen M. Niesters M. (2018). Averting Opioid-Induced Respiratory Depression without Affecting Analgesia. Anesthesiology 128, 1027–1037. 10.1097/ALN.0000000000002184 29553984

[B22] Davisson R. L. Travis M. D. Bates J. N. Johnson A. K. Lewis S. J. (1997). Stereoselective Actions of S-Nitrosocysteine in Central Nervous System of Conscious Rats. Am. J. Physiol. 272, H2361–H2368. 10.1152/ajpheart.1997.272.5.H2361 9176306

[B23] Davisson R. L. Travis M. D. Bates J. N. Lewis S. J. (1996). Hemodynamic Effects of L- and D-S-Nitrosocysteine in the Rat. Stereoselective S-Nitrosothiol Recognition Sites. Circ. Res. 79, 256–262. 10.1161/01.res.79.2.256 8756002

[B24] Deo V. S. Gilson T. P. Kaspar C. Singer M. E. (2021). The Fentanyl Phase of the Opioid Epidemic in Cuyahoga County, Ohio, United States. J. Forensic. Sci. 66, 926–933. 10.1111/1556-4029.14665 33394503

[B25] Eguchi K. Tadaki E. Simbulan D. Kumazawa T. (1987). Respiratory Depression Caused by Either Morphine Microinjection or Repetitive Electrical Stimulation in the Region of the Nucleus Parabrachialis of Cats. Pflugers Arch. 409, 367–373. 10.1007/BF00583790 3627956

[B26] Epstein M. A. Epstein R. A. (1978). A Theoretical Analysis of the Barometric Method for Measurement of Tidal Volume. Respir. Physiol. 32, 105–120. 10.1016/0034-5687(78)90103-2 625610

[B27] Epstein R. A. Epstein M. A. Haddad G. G. Mellins R. B. (1980). Practical Implementation of the Barometric Method for Measurement of Tidal Volume. J. Appl. Physiol. Respir. Environ. Exerc Physiol. 49, 1107–1115. 10.1152/jappl.1980.49.6.1107 7440298

[B28] Figueiredo V. C. Feksa L. R. Wannmacher C. M. (2009). Cysteamine Prevents Inhibition of Adenylate Kinase Caused by Cystine in Rat Brain Cortex. Metab. Brain Dis. 24, 373–381. 10.1007/s11011-009-9141-x10.1007/s11011-009-9142-9 19688256

[B29] Fone K. C. Wilson H. (1986). The Effects of Alfentanil and Selected Narcotic Analgesics on the Rate of Action Potential Discharge of Medullary Respiratory Neurones in Anaesthetized Rats. Br. J. Pharmacol. 89, 67–76. 10.1111/j.1476-5381.1986.tb11121.x 2879593 PMC1917050

[B30] Foreman J. W. Benson L. (1990). Effect of Cystine Loading and Cystine Dimethylester on Renal Brushborder Membrane Transport. Biosci. Rep. 10, 455–459. 10.1007/BF01152292 2282372

[B31] Gaston B. Baby S. M. May W. J. Young A. P. Grossfield A. Bates J. N. (2021). D-Cystine di(m)ethyl ester reverses the deleterious effects of morphine on ventilation and arterial blood gas chemistry while promoting antinociception. Sci. Rep. 11, 10038. 10.1038/s41598-021-89455-2 33976311 PMC8113454

[B32] Gaston B. May W. J. Sullivan S. Yemen S. Marozkina N. V. Palmer L. A. (2014). Essential Role of Hemoglobin Beta-93-Cysteine in Posthypoxia Facilitation of Breathing in Conscious Mice. J. Appl. Physiol. (1985) 116, 1290–1299. 10.1152/japplphysiol.01050.2013 24610531 PMC4044395

[B33] Gaston B. Smith L. Bosch J. Seckler J. Kunze D. Kiselar J. (2020). Voltage-gated Potassium Channel Proteins and Stereoselective S-Nitroso-L-Cysteine Signaling. JCI Insight 5, e134174. 10.1172/jci.insight.134174 PMC7526540 32790645

[B34] Getsy P. M. Coffee G. A. Lewis S. J. (2020). The Role of Carotid Sinus Nerve Input in the Hypoxic-Hypercapnic Ventilatory Response in Juvenile Rats. Front. Physiol. 11, 613786. 10.3389/fphys.2020.613786 33391030 PMC7773764

[B35] Getsy P. M. Davis J. Coffee G. A. May W. J. Palmer L. A. Strohl K. P. (2014). Enhanced Non-eupneic Breathing Following Hypoxic, Hypercapnic or Hypoxic-Hypercapnic Gas Challenges in Conscious Mice. Respir. Physiol. Neurobiol. 204, 147–159. 10.1016/j.resp.2014.09.006 25242462 PMC4252883

[B36] Getsy P. M. Sundararajan S. May W. J. von Schill G. C. McLaughlin D. K. Palmer L. A. (2021). Short-term Facilitation of Breathing upon Cessation of Hypoxic Challenge Is Impaired in Male but Not Female Endothelial NOS Knock-Out Mice. Sci. Rep. 11, 18346. 10.1038/s41598-021-97322-3 34526532 PMC8443732

[B37] Getsy P. M. Young A. P. Gaston B. Bates J. N. Baby S. M. Seckler J. M. (2022). S-Nitroso-L-Cysteine Stereoselectively Blunts the Negative Effects of Morphine on Breathing and Arterial Blood Gas Chemistry while Promoting Analgesia. Front. Pharmacol. in press. 10.1016/j.biopha.2022.113436 PMC9464305 36076552

[B38] Golder F. J. Dax S. Baby S. M. Gruber R. Hoshi T. Ideo C. (2015). Identification and Characterization of GAL-021 as a Novel Breathing Control Modulator. Anesthesiology 123, 1093–1104. 10.1097/ALN.0000000000000844 26352381

[B39] Gomez M. R. Benzick A. E. Rogers L. K. Heird W. C. Smith C. V. (1994). Attenuation of Acetaminophen Hepatotoxicity in Mice as Evidence for the Bioavailability of the Cysteine in D-Glucose-L-Cysteine *In Vivo* . Toxicol. Lett. 70, 101–108. 10.1016/0378-4274(94)90149-x 8310451

[B40] Granillo O. M. Brahmajothi M. V. Li S. Whorton A. R. Mason S. N. McMahon T. J. (2008). Pulmonary Alveolar Epithelial Uptake of S-Nitrosothiols Is Regulated by L-type Amino Acid Transporter. Am. J. Physiol. Lung Cell Mol. Physiol. 295, L38–L43. 10.1152/ajplung.00280.2007 18441097 PMC2494774

[B41] Grim T. W. Acevedo-Canabal A. Bohn L. M. (2020). Toward Directing Opioid Receptor Signaling to Refine Opioid Therapeutics. Biol. Psychiatry 87, 15–21. 10.1016/j.biopsych.2019.10.020 31806082 PMC6919561

[B42] Gurbuz N. Park M. A. Dent P. Abdel Mageed A. B. Sikka S. C. Baykal A. (2015). Cystine Dimethyl Ester Induces Apoptosis through Regulation of PKC-δ and PKC-ε in Prostate Cancer Cells. Anticancer Agents Med. Chem. 15, 217–227. 10.2174/1871520614666141120121901 25410184

[B43] Hamelmann E. Schwarze J. Takeda K. Oshiba A. Larsen G. L. Irvin C. G. (1997). Noninvasive Measurement of Airway Responsiveness in Allergic Mice Using Barometric Plethysmography. Am. J. Respir. Crit. Care Med. 156, 766–775. 10.1164/ajrccm.156.3.9606031 9309991

[B44] Hargreaves K. Dubner R. Brown F. Flores C. Joris J. (1988). A New and Sensitive Method for Measuring Thermal Nociception in Cutaneous Hyperalgesia. Pain 32, 77–88. 10.1016/0304-3959(88)90026-7 3340425

[B45] Hassen A. H. Feuerstein G. Pfeiffer A. Faden A. I. (1982). Delta versus Mu Receptors: Cardiovascular and Respiratory Effects of Opiate Agonists Microinjected into Nucleus Tractus Solitarius of Cats. Regul. Pept. 4, 299–309. 10.1016/0167-0115(82)90140-9 6294751

[B46] Hatfield M. J. Umans R. A. Hyatt J. L. Edwards C. C. Wierdl M. Tsurkan L. (2016). Carboxylesterases: General Detoxifying Enzymes. Chem. Biol. Interact. 259, 327–331. 10.1016/j.cbi.2016.02.011 26892220 PMC4985501

[B47] Henderson F. May W. J. Gruber R. B. Discala J. F. Puskovic V. Young A. P. (2014). Role of Central and Peripheral Opiate Receptors in the Effects of Fentanyl on Analgesia, Ventilation and Arterial Blood-Gas Chemistry in Conscious Rats. Respir. Physiol. Neurobiol. 191, 95–105. 10.1016/j.resp.2013.11.005 24284037 PMC4391496

[B48] Henderson F. May W. J. Gruber R. B. Young A. P. Palmer L. A. Gaston B. (2013). Low-dose Morphine Elicits Ventilatory Excitant and Depressant Responses in Conscious Rats: Role of Peripheral μ-opioid Receptors. Open J. Mol. Integr. Physiol. 3, 111–124. 10.4236/ojmip.2013.33017 24900948 PMC4041292

[B49] Henderson M. Rice B. Sebastian A. Sullivan P. G. King C. Robinson R. A. (2016). Neuroproteomic Study of Nitrated Proteins in Moderate Traumatic Brain Injured Rats Treated with Gamma Glutamyl Cysteine Ethyl Ester Administration Post Injury: Insight into the Role of Glutathione Elevation in Nitrosative Stress. Proteomics Clin. Appl. 10, 1218–1224. 10.1002/prca.201600004 27739215 PMC5698013

[B50] Hess D. T. Stamler J. S. (2012). Regulation by S-Nitrosylation of Protein Post-translational Modification. J. Biol. Chem. 287, 4411–4418. 10.1074/jbc.R111.285742 22147701 PMC3281651

[B51] Himi T. Ikeda M. Yasuhara T. Nishida M. Morita I. (2003). Role of Neuronal Glutamate Transporter in the Cysteine Uptake and Intracellular Glutathione Levels in Cultured Cortical Neurons. J. Neural Transm. (Vienna) 110, 1337–1348. 10.1007/s00702-003-0049-z 14666406

[B52] Hobbs M. J. Butterworth M. Cohen G. M. Upshall D. G. (1993). Structure-activity Relationships of Cysteine Esters and Their Effects on Thiol Levels in Rat Lung *In Vitro* . Biochem. Pharmacol. 45, 1605–1612. 10.1016/0006-2952(93)90301-c 8484801

[B53] Hosseini M. Taiarani Z. Hadjzadeh M. A. Salehabadi S. Tehranipour M. Alaei H. A. (2011). Different Responses of Nitric Oxide Synthase Inhibition on Morphine-Induced Antinociception in Male and Female Rats. Pathophysiology 18, 143–149. 10.1016/j.pathophys.2010.05.004 20558049

[B54] Iniushkin A. N. (1997). Respiratory and Hemodynamic Responses to Microinjections of Opioids into the Solitary Tract Nucleus. Ross. Fiziol. Zh. Im. I. M.Sechenova. 83, 112–121. 12436691

[B55] Ito L. Okumura M. Tao K. Kasai Y. Tomita S. Oosuka A. (2012). Glutathione Ethylester, a Novel Protein Refolding Reagent, Enhances Both the Efficiency of Refolding and Correct Disulfide Formation. Protein J. 31, 499–503. 10.1007/s10930-012-9427-4 22752753

[B56] Jenkins M. W. Khalid F. Baby S. M. May W. J. Young A. P. Bates J. N. (2021). Glutathione Ethyl Ester Reverses the Deleterious Effects of Fentanyl on Ventilation and Arterial Blood-Gas Chemistry while Prolonging Fentanyl-Induced Analgesia. Sci. Rep. 11, 6985. 10.1038/s41598-021-86458-x 33772077 PMC7997982

[B57] Kasaba T. Takeshita M. Takasaki M. (1997). The Effects of Caffeine on the Respiratory Depression by Morphine. Masui 46, 1570–1574. 9455078

[B58] Kimura H. (2014). The Physiological Role of Hydrogen Sulfide and beyond. Nitric Oxide 41, 4–10. 10.1016/j.niox.2014.01.002 24491257

[B59] Lailey A. F. Hill L. Lawston I. W. Stanton D. Upshall D. G. (1991). Protection by Cysteine Esters against Chemically Induced Pulmonary Oedema. Biochem. Pharmacol. 42 (Suppl. l), S47–S54. 10.1016/0006-2952(91)90391-h 1768285

[B60] Lailey A. F. Upshall D. G. (1994). Thiol Levels in Rat Bronchio-Alveolar Lavage Fluid after Administration of Cysteine Esters. Hum. Exp. Toxicol. 13, 776–780. 10.1177/096032719401301106 7857697

[B61] Lalley P. M. Pilowsky P. M. Forster H. V. Zuperku E. J. (2014). CrossTalk Opposing View: the Pre-botzinger Complex Is Not Essential for Respiratory Depression Following Systemic Administration of Opioid Analgesics. J. Physiol. 592, 1163–1166. 10.1113/jphysiol.2013.258830 24634012 PMC3961072

[B62] Lewis S. J. Hoque A. Bates J. N. (2005). Differentiation of L- and D-S-Nitrosothiol Recognition Sites *In Vivo* . J. Cardiovasc. Pharmacol. 46, 660–671. 10.1097/01.fjc.0000181714.94827.5d 16220074

[B63] Lewis S. J. Owen J. R. Bates J. N. (2006). S-nitrosocysteine Elicits Hemodynamic Responses Similar to Those of the Bezold-Jarisch Reflex via Activation of Stereoselective Recognition Sites. Eur. J. Pharmacol. 531, 254–258. 10.1016/j.ejphar.2005.11.027 16438953

[B64] Lewis S. J. Travis M. D. Bates J. N. (1996). Stereoselective S-Nitrosocysteine Recognition Sites in Rat Brain. Eur. J. Pharmacol. 312, R3–R5. 10.1016/0014-2999(96)00607-3 8894607

[B65] Lewis S. J. Meller S. T. Brody M. J. Gebhart G. F. (1991). Reduced Nociceptive Effects of Intravenous Serotonin (5-HT) in the Spontaneously Hypertensive Rat. Clin. Exp. Hypertens. Part A Theory Pract. 13, 849–857. 10.3109/10641969109042089 1773517

[B66] Li S. Whorton A. R. (2007). Functional Characterization of Two S-Nitroso-L-Cysteine Transporters, Which Mediate Movement of NO Equivalents into Vascular Cells. Am. J. Physiol. Cell Physiol. 292, C1263–C1271. 10.1152/ajpcell.00382.2006 17092994

[B67] Li W. M. Sato A. Sato Y. Schmidt R. F. (1996). Morphine Microinjected into the Nucleus Tractus Solitarius and Rostral Ventrolateral Medullary Nucleus Enhances Somatosympathetic A- and C- Reflexes in Anesthetized Rats. Neurosci. Lett. 221, 53–56. 10.1016/s0304-3940(96)13286-9 9014179

[B68] Li Y. Su L. Li F. Wang C. Yuan D. Chen J. (20152015). Acute and Sub-chronic Toxicity of Glucose-Cysteine Maillard Reaction Products in Sprague-Dawley Rats. Food Chem. Toxicol. 80, 271–276. 10.1016/j.fct.2015.03.021 25817020

[B69] Lipton A. J. Johnson M. A. Macdonald T. Lieberman M. W. Gozal D. Gaston B. (2001). S-nitrosothiols Signal the Ventilatory Response to Hypoxia. Nature 413, 171–174. 10.1038/35093117 11557982

[B70] Lomask M. (2006). Further Exploration of the Penh Parameter. Exp. Toxicol. Pathol. 5 (2), 13–20. 10.1016/j.etp.2006.02.014 16638630

[B71] Ludbrook J. (1998). Multiple Comparison Procedures Updated. Clin. Exp. Pharmacol. Physiol. 25, 1032–1037. 10.1111/j.1440-1681.1998.tb02179.x 9888002

[B72] May W. J. Gruber R. B. Discala J. F. Puskovic V. Henderson F. Palmer L. A. (2013b). Morphine Has Latent Deleterious Effects on the Ventilatory Responses to a Hypoxic Challenge. Open J. Mol. Integr. Physiol. 3, 166–180. 10.4236/ojmip.2013.34022 25045593 PMC4103751

[B73] May W. J. Henderson F. Gruber R. B. Discala J. F. Young A. P. Bates J. N. (2013a). Morphine Has Latent Deleterious Effects on the Ventilatory Responses to a Hypoxic-Hypercapnic Challenge. Open J. Mol. Integr. Physiol. 3, 134–145. 10.4236/ojmip.2013.33019 25045592 PMC4103749

[B74] McHugh M. L. (2011). Multiple Comparison Analysis Testing in ANOVA. Biochem. Med. Zagreb. 21, 203–209. 10.11613/bm.2011.029 22420233

[B75] Meller S. T. Lewis S. J. Brody M. J. Gebhart G. F. (1991). The Peripheral Nociceptive Actions of Intravenously Administered 5-HT in the Rat Requires Dual Activation of Both 5-HT2 and 5-HT3 Receptor Subtypes. Brain Res. 561, 61–68. 10.1016/0006-8993(91)90749-l 1797350

[B76] Mendoza J. Passafaro R. Baby S. Young A. P. Bates J. N. Gaston B. (2013). L-cysteine Ethyl Ester Reverses the Deleterious Effects of Morphine on, Arterial Blood-Gas Chemistry in Tracheotomized Rats. Respir. Physiol. Neurobiol. 189, 136–143. 10.1016/j.resp.2013.07.007 23892097 PMC4430552

[B77] Nemoto T. Shimma N. Horie S. Saito T. Okuma Y. Nomura Y. (2003). Involvement of the System L Amino Acid Transporter on Uptake of S-Nitroso-L-Cysteine, an Endogenous S-Nitrosothiol, in PC12 Cells. Eur. J. Pharmacol. 458, 17–24. 10.1016/s0014-2999(02)02699-7 12498902

[B78] Ohta H. Bates J. N. Lewis S. J. Talman W. T. (1997). Actions of S-Nitrosocysteine in the Nucleus Tractus Solitarii Are Unrelated to Release of Nitric Oxide. Brain Res. 746, 98–104. 10.1016/s0006-8993(96)01188-2 9037488

[B79] Palmer L. A. Kimberly deRonde K. Brown-Steinke K. Gunter S. Jyothikumar V. Forbes M. S. (2015). Hypoxia-induced Changes in Protein S-Nitrosylation in Female Mouse Brainstem. Am. J. Respir. Cell. Mol. Biol. 52, 37–45. 10.1165/rcmb.2013-0359OC 24922346 PMC4370248

[B80] Palmer L. A. May W. J. deRonde K. Brown-Steinke K. Bates J. N. Gaston B. (2013). Ventilatory Responses during and Following Exposure to a Hypoxic Challenge in Conscious Mice Deficient or Null in S-Nitrosoglutathione Reductase. Respir. Physiol. Neurobiol. 185, 571–581. 10.1016/j.resp.2012.11.009 23183419 PMC3593598

[B81] Pathirathna S. Covey D. F. Todorovic S. M. Jevtovic-Todorovic V. (2006). Differential Effects of Endogenous Cysteine Analogs on Peripheral Thermal Nociception in Intact Rats. Pain 125, 53–64. 10.1016/j.pain.2006.04.024 16782275

[B82] Paul B. D. Sbodio J. I. Snyder S. H. (2018). Cysteine Metabolism in Neuronal Redox Homeostasis. Trends Pharmacol. Sci. 39, 513–524. 10.1016/j.tips.2018.02.007 29530337 PMC5912966

[B83] Perissinotti L. L. Turjanski A. G. Estrin D. A. Doctorovich F. (20052005). Transnitrosation of Nitrosothiols: Characterization of an Elusive Intermediate. J. Am. Chem. Soc. 127 (2), 486–487. 10.1021/ja044056v 15643848

[B84] Quindry J. C. Ballmann C. G. Epstein E. E. Selsby J. T. (2016). Plethysmography Measurements of Respiratory Function in Conscious Unrestrained Mice. J. Physiol. Sci. 66, 157–164. 10.1007/s12576-015-0408-1 26459291 PMC10717823

[B85] Rech V. C. Feksa L. R. Fleck R. M. Athaydes G. A. Dornelles P. K. Rodrigues-Junior V. (2008). Cysteamine Prevents Inhibition of Thiol-Containing Enzymes Caused by Cystine or Cystine Dimethylester Loading in Rat Brain Cortex. Metab. Brain Dis. 23, 133–145. 10.1007/s11011-008-9081-x 18418703

[B86] Ren J. Ding X. Greer J. J. (20152015). 5-HT1A Receptor Agonist Befiradol Reduces Fentanyl-Induced Respiratory Depression, Analgesia, and Sedation in Rats. Anesthesiology 122, 424–434. 10.1097/ALN.0000000000000490 25313880

[B87] Ren J. Ding X. Greer J. J. (2020). Countering Opioid-Induced Respiratory Depression in Male Rats with Nicotinic Acetylcholine Receptor Partial Agonists Varenicline and ABT 594. Anesthesiology 132, 1197–1211. 10.1097/ALN.0000000000003128 32294065

[B88] Satoh T. Hosokawa M. (2006). Structure, Function and Regulation of Carboxylesterases. Chem. Biol. Interact. 162, 195–211. 10.1016/j.cbi.2006.07.001 16919614

[B89] Satoh T. Hosokawa M. (1998). The Mammalian Carboxylesterases: from Molecules to Functions. Annu. Rev. Pharmacol. Toxicol. 38, 257–288. 10.1146/annurev.pharmtox.38.1.257 9597156

[B90] Schmid C. L. Kennedy N. M. Ross N. C. Lovell K. M. Yue Z. Morgenweck J. (2017). Bias Factor and Therapeutic Window Correlate to Predict Safer Opioid Analgesics. Cell 171, 1165–e13. e13. 10.1016/j.cell.2017.10.035 29149605 PMC5731250

[B91] Seckler J. M. Grossfield A. May W. J. Getsy P. M. Lewis S. J. (2022). Nitrosyl Factors Play a Vital Role in the Ventilatory Depressant Effects of Fentanyl in Unanesthetized Rats. Biomed. Pharmacother. 146, 112571. 10.1016/j.biopha.2021.112571 34953397 PMC8776621

[B92] Seckler J. M. Lewis S. J. (2020). Advances in D-Amino Acids in Neurological Research. Int. J. Mol. Sci. 21, 7325. 10.3390/ijms21197325 PMC7582301 33023061

[B93] Seckler J. M. Meyer N. M. Burton S. T. Bates J. N. Gaston B. Lewis S. J. (2017). Detection of Trace Concentrations of S-Nitrosothiols by Means of a Capacitive Sensor. PLoS One 12, e0187149. 10.1371/journal.pone.0187149 29073241 PMC5658150

[B94] Seckler J. M. Shen J. Lewis T. H. J. Abdulameer M. A. Zaman K. Palmer L. A. (2020). NADPH Diaphorase Detects S-Nitrosylated Proteins in Aldehyde-Treated Biological Tissues. Sci. Rep. 10, 21088. 10.1038/s41598-020-78107-6 33273578 PMC7713249

[B95] Semenza E. R. Harraz M. M. Abramson E. Malla A. P. Vasavda C. Gadalla M. M. (2021). D-cysteine Is an Endogenous Regulator of Neural Progenitor Cell Dynamics in the Mammalian Brain. Proc. Natl. Acad. Sci. U. S. A. 118, e2110610118. 10.1073/pnas.2110610118 34556581 PMC8488618

[B96] Servin A. L. Goulinet S. Renault H. (1988). Pharmacokinetics of Cysteine Ethyl Ester in Rat. Xenobiotica 18, 839–847. 10.3109/00498258809041722 3176522

[B97] Shanker G. Aschner M. (2001). Identification and Characterization of Uptake Systems for Cystine and Cysteine in Cultured Astrocytes and Neurons: Evidence for Methylmercury-Targeted Disruption of Astrocyte Transport. J. Neurosci. Res. 66, 998–1002. 10.1002/jnr.10066 11746429

[B98] Shibuya N. Kimura H. (2013). Production of Hydrogen Sulfide from D-Cysteine and its Therapeutic Potential. Front. Endocrinol. (Lausanne) 4, 87. 10.3389/fendo.2013.00087 23882260 PMC3712494

[B99] Simmons-Willis T. A. Koh A. S. Clarkson T. W. Ballatori N. (2002). Transport of a Neurotoxicant by Molecular Mimicry: the Methylmercury-L-Cysteine Complex Is a Substrate for Human L-type Large Neutral Amino Acid Transporter (LAT) 1 and LAT2. Biochem. J. 367, 239–246. 10.1042/BJ20020841 12117417 PMC1222880

[B100] Stein P. D. Goldhaber S. Z. Henry J. W. (1995). Alveolar-arterial Oxygen Gradient in the Assessment of Acute Pulmonary Embolism. Chest 107, 139–143. 10.1378/chest.107.1.139 7632205

[B101] Stengel A. Coskun T. Goebel M. Wang L. Craft L. Alsina-Fernandez J. (2010). Central Injection of the Stable Somatostatin Analog ODT8-SST Induces a Somatostatin2 Receptor-Mediated Orexigenic Effect: Role of Neuropeptide Y and Opioid Signaling Pathways in Rats. Endocrinology 151, 4224–4235. 10.1210/en.2010-0195 20610566 PMC2940496

[B102] Stomberski C. T. Hess D. T. Stamler J. S. (2019). Protein S-Nitrosylation: Determinants of Specificity and Enzymatic Regulation of S-Nitrosothiol-Based Signaling. Antioxid. Redox Signal. 30, 1331–1351. 10.1089/ars.2017.7403 29130312 PMC6391618

[B103] Story D. A. (1996). Alveolar Oxygen Partial Pressure, Alveolar Carbon Dioxide Partial Pressure, and the Alveolar Gas Equation. Anesthesiology 84, 1011. 10.1097/00000542-199604000-00036 8638826

[B104] Sumayao R. McEvoy B. Martin-Martin N. McMorrow T. Newsholme P. (2013). Cystine Dimethylester Loading Promotes Oxidative Stress and a Reduction in ATP Independent of Lysosomal Cystine Accumulation in a Human Proximal Tubular Epithelial Cell Line. Exp. Physiol. 98, 1505–1517. 10.1113/expphysiol.2013.073809 23813804

[B105] Szwergold B. S. (2006). Alpha-thiolamines Such as Cysteine and Cysteamine Act as Effective Transglycating Agents Due to Formation of Irreversible Thiazolidine Derivatives. Med. Hypotheses 66, 698–707. 10.1016/j.mehy.2005.10.029 16359826

[B106] Todorovic S. M. Jevtovic-Todorovic V. (2014). Redox Regulation of Neuronal Voltage-Gated Calcium Channels. Antioxid. Redox Signal. 21, 880–891. 10.1089/ars.2013.5610 24161125 PMC4116091

[B107] Travis M. D. Davisson R. L. Bates J. N. Lewis S. J. (1997). Hemodynamic Effects of L- and D-S-Nitroso-Beta,beta-Dimethylcysteine in Rats. Am. J. Physiol. 273, H1493–H1501. 10.1152/ajpheart.1997.273.3.H1493 9321842

[B108] Travis M. D. Stoll L. L. Bates J. N. Lewis S. J. (1996). L- and D-S-Nitroso-Beta,beta-Dimethylcysteine Differentially Increase cGMP in Cultured Vascular Smooth Muscle Cells. Eur. J. Pharmacol. 318, 47–53. 10.1016/s0014-2999(96)00719-4 9007512

[B109] Trivedi M. Shah J. Hodgson N. Byun H. M. Deth R. (2014). Morphine Induces Redox-Based Changes in Global DNA Methylation and Retrotransposon Transcription by Inhibition of Excitatory Amino Acid Transporter Type 3-mediated Cysteine Uptake. Mol. Pharmacol. 85, 747–757. 10.1124/mol.114.091728 24569088 PMC3990020

[B110] Trivedi M. S. Deth R. (2015). Redox-based Epigenetic Status in Drug Addiction: a Potential Contributor to Gene Priming and a Mechanistic Rationale for Metabolic Intervention. Front. Neurosci. 8, 444. 10.3389/fnins.2014.00444 25657617 PMC4302946

[B111] Tsikas D. Schwedhelm K. S. Surdacki A. Giustarini D. Rossi R. Kukoc-Modun L. (2018). S-Nitroso-N-acetyl-L-cysteine Ethyl Ester (SNACET) and N-Acetyl-L-Cysteine Ethyl Ester (NACET)-Cysteine-based Drug Candidates with Unique Pharmacological Profiles for Oral Use as NO, H2S and GSH Suppliers and as Antioxidants: Results and Overview. J. Pharm. Anal. 8, 1–9. 10.1016/j.jpha.2017.12.003 29568662 PMC5859134

[B112] Tsumuro T. Alejandra Hossen M. Kishi Y. Fujii Y. Kamei C. (2006). Nasal Congestion Model in Brown Norway Rats and the Effects of Some H1-Antagonists. Int. Immunopharmacol. 6, 759–763. 10.1016/j.intimp.2005.11.009 16546706

[B113] Tunnicliff G. (1994). Amino Acid Transport by Human Erythrocyte Membranes. Comp. Biochem. Physiol. Comp. Physiol. 108, 471–478. 10.1016/0300-9629(94)90329-8 7915653

[B114] van Dorp E. Yassen A. Dahan A. (2007). Naloxone Treatment in Opioid Addiction: the Risks and Benefits. Expert Opin. Drug Saf. 6, 125–132. 10.1517/14740338.6.2.125 17367258

[B115] Varga A. G. Reid B. T. Kieffer B. L. Levitt E. S. (2020). Differential Impact of Two Critical Respiratory Centres in Opioid-Induced Respiratory Depression in Awake Mice. J. Physiol. 598, 189–205. 10.1113/JP278612 31589332 PMC6938533

[B116] Wallenstein S. Zucker C. L. Fleiss J. L. (1980). Some Statistical Methods Useful in Circulation Research. Circ. Res. 47, 1–9. 10.1161/01.res.47.1.1 7379260

[B117] Williams F. M. (1985). Clinical Significance of Esterases in Man. Clin. Pharmacokinet. 10, 392–403. 10.2165/00003088-198510050-00002 3899454

[B118] Williams J. T. Ingram S. L. Henderson G. Chavkin C. von Zastrow M. Schulz S. (2013). Regulation of μ-opioid Receptors: Desensitization, Phosphorylation, Internalization, and Tolerance. Pharmacol. Rev. 65, 223–254. 10.1124/pr.112.00594243 23321159 PMC3565916

[B119] Wróbel M. Ubuka T. Yao W. B. Abe T. (1997). Effect of Glucose-Cysteine Adduct on Cysteine Desulfuration in guinea Pig Tissues. Physiol. Chem. Phys. Med. NMR 29, 11–14. 9353953

[B120] Wu G. (2009). Amino Acids: Metabolism, Functions, and Nutrition. Amino Acids 37, 1–17. 10.1007/s00726-009-0269-0 19301095

[B121] Yin J. Ren W. Yang G. Duan J. Huang X. Fang R. (2016). L-cysteine Metabolism and its Nutritional Implications. Mol. Nutr. Food Res. 60, 134–146. 10.1002/mnfr.201500031 25929483

[B122] Young A. P. Gruber R. B. Discala J. F. May W. J. Palmer L. A. Lewis S. J. (2013). Co-activation of μ- and δ-opioid Receptors Elicits Tolerance to Morphine-Induced Ventilatory Depression via Generation of Peroxynitrite. Resp. Physiol. Neurobiol. 186, 255–264. 10.1016/j.resp.2013.02.028 PMC4451624 23473921

[B123] Young J. D. Jones S. E. Ellory J. C. (1981). Amino Acid Transport via the Red Cell Anion Transport System. Biochim. Biophys. Acta 645, 157–160. 10.1016/0005-2736(81)90524-1 6789878

[B124] Yu C. Yuan M. Yang H. Zhuang X. Li H. (2018). P-glycoprotein on Blood-Brain Barrier Plays a Vital Role in Fentanyl Brain Exposure and Respiratory Toxicity in Rats. Toxicol. Sci. 164, 353–362. 10.1093/toxsci/kfy093 29669042

[B125] Zerangue N. Kavanaugh M. P. (1996). Interaction of L-Cysteine with a Human Excitatory Amino Acid Transporter. J. Physiol. 493 ( Pt 2), 419–423. 10.1113/jphysiol.1996.sp021393 8782106 PMC1158927

[B126] Zhuang J. Zhang Z. Zhang C. Xu F. (2012). 8-OH-DPAT Abolishes the Pulmonary C-Fiber-Mediated Apneic Response to Fentanyl Largely via Acting on 5HT1A Receptors in the Nucleus Tractus Solitarius. Am. J. Physiol. Regul. Integr. Comp. Physiol. 303, R449–R458. 10.1152/ajpregu.00016.2012 22696579 PMC3423994

