# Supplementary material for: D-Cysteine Ethyl Ester Reverses the Deleterious Effects of Morphine on Breathing and Arterial Blood–Gas Chemistry in Freely-Moving Rats
Source: Front Pharmacol. 2022 Jun 23;13:883329. doi: 10.3389/fphar.2022.883329 (PMC9260251; doi:10.3389/fphar.2022.883329)
Supplement: Supplementary file 1 [file DataSheet1.docx]

**Supplementary Material**

**D-Cysteine Ethyl Ester Reverses the Deleterious Effects of Morphine on Breathing**

**and Arterial Blood-Gas Chemistry in Freely-Moving Rats**

Paulina M. Getsy,^1^* Santhosh M. Baby,^2,†^ Walter J. May,^3^ Alex P. Young,^3^

Benjamin Gaston,^4^ Matthew R. Hodges,^5^ Hubert V. Forster,^5^ James N. Bates,^6^

Christopher G. Wilson,^7^ Tristan H. J. Lewis,^1^ Yee-Hee Hsieh,^8^ Stephen J. Lewis,^1,9^

*^1^Department of Pediatrics, Case Western Reserve University, Cleveland, OH 44106, USA*

*^2^Department of Drug Discovery, Galleon Pharmaceuticals, Inc., Horsham, PA 19044, USA*

*^3^Pediatric Respiratory Medicine, University of Virginia School of Medicine,*

*Charlottesville, VA 22908, USA*

*^4^Herman B Wells Center for Pediatric Research, Indiana University School of Medicine*

*Indianapolis, IN 46202, USA*

*^5^Department of Physiology, Medical College of Wisconsin, Milwaukee, WI 53226, USA*

*^6^Department of Anesthesia, University of Iowa Hospitals and Clinics, Iowa City, IA 52242, USA*

*^7^Basic Sciences, Division of Physiology, School of Medicine, Loma Linda University*

*Loma Linda, CA 92350, USA*

*^8^Division of Pulmonary, Critical Care and Sleep Medicine, University Hospitals Case Medical Center, Case Western Reserve University, Cleveland, OH, OH 44106, USA*

*^9^Department of Pharmacology, Case Western Reserve University, Cleveland, Ohio 44106, USA*

***Corresponding Author:** Paulina M. Getsy, PhD. Department of Pediatrics, Division of Pulmonology, Allergy and Immunology, School of Medicine, Case Western Reserve University, 10900 Euclid Avenue, Cleveland, OH 44106-4984. Email: pxg55@case.edu

**^†^Current address:** Santhosh M. Baby, Translational Sciences Treatment Discovery, Galvani Bioelectronics, Inc., 1250 S Collegeville Rd., Collegeville, Pennsylvania 19426. Email: santhosh.m.baby@galvani.bio

**Supplementary Table S1**

Definition of ventilatory parameters described in this study

| **Parameter** | **Abbreviation** | | **Units** | | **Definition** |
| --- | --- | --- | --- | --- | --- |
| **A. Directly recorded parameters** | | | | | |
| Frequency of Breaths | | Freq | | breaths/min | Rate of breathing |
| Inspiratory Time | | Ti | | sec | Duration of inspiration |
| Expiratory Time | | Te | | sec | Duration of expiration |
| End Inspiratory Pause | | EIP | | msec | Pause between end of inspiration start of expiration |
| End Expiratory Pause | | EEP | | msec | Pause between end of expiration and start of inspiration |
| Relaxation Time | | RT | | sec | Decay of expiration to 36% maximum |
| Tidal Volume | | TV | | ml | Volume of inspired air per breath |
| Peak Inspiratory Flow | | PIF | | ml/sec | Maximum inspiratory flow |
| Peak Expiratory Flow | | PEF | | ml/sec | Maximum expiratory flow |
| Expiratory flow at 50% | | EF_50_ | | ml/sec | Expiratory flow at 50% expired TV |
| Non-Eupneic Breathing Index | | NEBI | | % | % of non-eupneic breaths per epoch |
| **B. Derived parameters** | | | | | |
| Minute Ventilation | | MV = freq x TV | | ml/min | Total volume of air inspired per min |
| Ti/Te | | Ti/Te | | none | Inspiratory quotient |
| PEF/PIF | | PEF/PIF | | none | Flow balance |
| Apneic Pause | | AP = (Te/RT)-1 | | No units | Elongated expiration |
| Expiratory Delay | | Te-RT | | No units | Difference in lengths of Te and RT |
| Inspiratory Drive | | TV/Ti | | ml/sec | Central urge to inhale |
| Expiratory Drive | | TV/Te | | ml/sec | Central drive to exhale |
| NEBI/Frequency | | NEBI/Freq | | %/(b/min) | Balanced rejection index |

**Supplementary Figure S1**

**Supplementary Figure S1.** Relationships between peak inspiratory flow (PIF), peak expiratory flow (PEF), relaxation time (RT) and expiratory time (Te).

**Supplementary Table S2**

Baseline (Pre) values in the groups of rats that would receive vehicle or D-cysteine ethyl ester

| **Parameter** |  | **Vehicle** |  | **D-CYSee** |
| --- | --- | --- | --- | --- |
| Frequency, breaths/min |  | 105 ± 3 |  | 103 ± 5 |
| Tidal Volume (TV), ml |  | 2.54 ± 0.13 |  | 2.55 ± 0.12 |
| Minute Ventilation, ml/min |  | 267 ± 16 |  | 262 ± 15 |
| Inspiratory Time (Ti), sec |  | 0.238 ± 0.006 |  | 0.236 ± 0.011 |
| Expiratory Time (Te), sec |  | 0.429 ± 0.012 |  | 0.421 ± 0.028 |
| Expiratory Time/Inspiratory Time |  | 1.81 ± 0.05 |  | 1.78 ± 0.08 |
| End Inspiratory Pause, msec |  | 7.15 ± 0.31 |  | 7.79 ± 0.26 |
| End Expiratory Pause, msec |  | 16.7 ± 0.34 |  | 17.2 ± 0.83 |
| Peak Inspiratory Flow, ml/sec |  | 16.0 ± 0.6 |  | 16.8 ± 1.3 |
| Peak Expiratory Flow, ml/sec |  | 11.3 ± 0.3 |  | 11.4 ± 0.6 |
| Peak Expiratory Flow/Peak Inspiratory Flow |  | 0.71 ± 0.04 |  | 0.70 ± 0.03 |
| EF_50_, ml/sec |  | 0.31 ± 0.02 |  | 0.33 ± 0.01 |
| Relaxation Time, sec |  | 0.27 ± 0.01 |  | 0.26 ± 0.02 |
| Apneic Pause, (Te/RT)-1 |  | 0.62 ± 0.04 |  | 0.64 ± 0.03 |
| Expiratory Delay, Te-RT |  | 0.163 ± 0.010 |  | 0.164 ± 0.015 |
| Inspiratory Drive (TV/Ti), ml/sec |  | 10.7 ± 0.5 |  | 10.8 ± 0.5 |
| Inspiratory Drive (TV/Te), ml/sec |  | 5.9 ± 0.3 |  | 6.2 ± 0.4 |
| Non-Eupneic Breathing Index (NEBI), % |  | 4.6 ± 0.7 |  | 5.6 ± 0.7 |
| NEBI/Frequency, %/(breaths/min) |  | 4.68 ± 0.60 |  | 5.52 ± 0.71 |

D-CYSee, D-cysteine ethyl ester. The data are presented as mean ± SEM. There were no between group differences for any parameter (P > 0.05, for all comparisons).

**Supplementary Table S3**

Morphine-induced ventilatory responses in rats that subsequently received vehicle or D-CYSee

|  |  | **+Peak Responses (%change)** | | |  | **-Peak Responses (%change)** | | |
| --- | --- | --- | --- | --- | --- | --- | --- | --- |
| **Parameters** |  | **Vehicle** |  | **D-CYSee** |  | **Vehicle** |  | **D-CYSee** |
| Frequency, breaths/min |  | +10 ± 3* |  | +11 ± 4* |  | -32 ± 3* |  | -29 ± 4* |
| Tidal Volume (TV), ml |  | -19 ± 6* |  | -22 ± 5* |  | -39 ± 5* |  | -48 ± 3* |
| Minute Ventilation, ml/min |  | -16 ± 4* |  | -18 ± 6* |  | -56 ± 4* |  | -60 ± 4* |
| Inspiratory Time (Ti), sec |  | -16 ± 2* |  | -20 ± 4* |  | +44 ± 3* |  | +43 ± 7* |
| Expiratory Time (Te), sec |  | +5 ± 6 |  | +1 ± 5 |  | +1 ± 6 |  | -4 ± 3 |
| Expiratory Time/Inspiratory Time |  | +18 ± 3* |  | +15 ± 5* |  | -30 ± 3* |  | -25 ± 3* |
| End Inspiratory Pause, msec |  | -15 ± 3* |  | -18 ± 4* |  | +128 ± 13* |  | +121 ± 15* |
| End Expiratory Pause, msec |  | +414 ± 47* |  | +390 ± 32* |  | +226 ± 19* |  | +225 ± 25* |
| Peak Inspiratory Flow, ml/sec |  | +27 ± 3* |  | +34 ± 5* |  | -39 ± 6* |  | -45 ± 4* |
| Peak Expiratory Flow, ml/sec |  | +50 ± 10* |  | +51 ± 12* |  | -26 ± 4* |  | -31 ± 5* |
| Peak Expiratory Flow/Peak Inspiratory Flow |  | +18 ± 6* |  | +13 ± 4* |  | +22 ± 3* |  | +26 ± 5* |
| EF_50_, ml/sec |  | +104 ± 5* |  | +92 ± 14* |  | +14 ± 2* |  | +12 ± 3* |
| Relaxation Time (RT), sec |  | -37 ± 4* |  | -43 ± 7* |  | -19 ± 4* |  | -16 ± 3* |
| Apneic Pause, (Te/RT)-1 |  | +162 ± 17* |  | +184 ± 20* |  | +93 ± 13* |  | +89 ± 12* |
| Expiratory Delay, Te-RT |  | +79 ± 18* |  | +51 ± 14* |  | +45 ± 16* |  | +48 ± 13* |
| Inspiratory Drive (TV/Ti), ml/sec |  | -8 ± 6 |  | -8 ± 7 |  | -51 ± 5* |  | -54 ± 5* |
| Expiratory Drive (TV/Te), ml/sec |  | -25 ± 6* |  | -19 ± 4* |  | -49 ± 6* |  | -53 ± 6* |
| Non-Eupneic Breathing Index (NEBI), % |  | +201 ± 14* |  | +203 ± 21* |  | +266 ± 21* |  | +285 ± 30* |
| NEBI/Frequency, %/(breaths/min) |  | +189 ± 11* |  | +176 ± 12* |  | +519 ± 23* |  | +483 ± 27* |

D-CYSee, D-cysteine ethyl ester (500 μmol/kg, IV). The data are presented as mean ± SEM. There were 5 rats in the vehicle group and 6 rats in the D-CYSee group. There were no between group differences for any parameter (P > 0.05, for all comparisons. *P < 0.05, D-CYSee *versus* vehicle.

**Supplementary Table S4**

Morphine-induced ventilatory responses in rats that subsequently received vehicle or D-CYSee

|  |  | **Values at +15 min (%change)** | | |  | **Total Response (% change)** | | |
| --- | --- | --- | --- | --- | --- | --- | --- | --- |
| **Parameters** |  | **Vehicle** |  | **D-CYSee** |  | **Vehicle** |  | **D-CYSee** |
| Frequency, breaths/min |  | -15 ± 3* |  | -14 ± 3* |  | -21 ± 4* |  | -18 ± 3* |
| Tidal Volume (TV), ml |  | -21 ± 2* |  | -22 ± 2* |  | -22 ± 3* |  | -26 ± 3* |
| Minute Ventilation, ml/min |  | -33 ± 4* |  | -32 ± 5* |  | -39 ± 3* |  | -40 ± 6* |
| Inspiratory Time (Ti), sec |  | +48 ± 7* |  | +47 ± 9* |  | +42 ± 6* |  | +39 ± 8* |
| Expiratory Time (Te), sec |  | -20 ± 2* |  | -22 ± 2* |  | -8 ± 6 |  | -7 ± 5 |
| Expiratory Time/Inspiratory Time |  | -47 ± 4* |  | -45 ± 3* |  | -33 ± 2* |  | -30 ± 3* |
| End Inspiratory Pause, msec |  | +154 ± 20* |  | +143 ± 16* |  | +122 ± 8* |  | +106 ± 13* |
| End Expiratory Pause, msec |  | -28 ± 7* |  | -27 ± 8* |  | +106 ± 12* |  | +105 ± 13* |
| Peak Inspiratory Flow, ml/sec |  | -32 ± 5* |  | -33 ± 5* |  | -29 ± 4* |  | -33 ± 6* |
| Peak Expiratory Flow, ml/sec |  | -3 ± 5 |  | +2 ± 6 |  | -8 ± 5 |  | -9 ± 9 |
| Peak Expiratory Flow/Peak Inspiratory Flow |  | +49 ± 7* |  | +53 ± 6* |  | +30 ± 3* |  | +38 ± 6* |
| EF_50_, ml/sec |  | +92 ± 12* |  | +84 ± 7* |  | +61 ± 12* |  | +54 ± 10* |
| Relaxation Time (RT), sec |  | -37 ± 6* |  | -35 ± 2* |  | -31 ± 3* |  | -29 ± 2* |
| Apneic Pause |  | +56 ± 9* |  | +50 ± 8* |  | +89 ± 10* |  | +78 ± 6* |
| Expiratory Delay, Te-RT |  | +3 ± 4 |  | -2 ± 3 |  | +31 ± 7* |  | +28 ± 8* |
| Inspiratory Drive (TV/Ti), ml/sec |  | -46 ± 2* |  | -45 ± 5* |  | -44 ± 2* |  | -46 ± 5* |
| Expiratory Drive (TV/Te), ml/sec |  | +5 ± 8 |  | +1 ± 6 |  | -14 ± 3* |  | -18 ± 4* |
| Non-Eupneic Breathing Index (NEBI), % |  | -40 ± 7* |  | -48 ± 6* |  | +74 ± 19* |  | +76 ± 20* |
| NEBI/Frequency (%/breaths/min) |  | -34 ± 7* |  | -40 ± 8* |  | +134 ± 17* |  | +122 ± 23* |

D-CYSee, D-cysteine ethyl ester (500 μmol/kg, IV). The data are presented as mean ± SEM. There were 5 rats in the vehicle group and 6 rats in the D-CYSee group. There were no between group differences for any parameter (P > 0.05, for all comparisons. *P < 0.05, D-CYSee *versus* vehicle.

**Supplementary Figure S2**

**A.**

**B.**

**Supplementary Figure S2.** Values of end inspiratory pause (**Panel A**) and end expiratory pause (**Panel B**) in freely-moving rats prior to (Pre) and following the injection of morphine (10 mg/kg, IV) and subsequent injections of vehicle (saline, IV) or D-cysteine ethyl ester (D-CYSee, 500 μmol/kg, IV). The data are presented as mean ± SEM. There were 5 rats in the vehicle group and 6 rats in the D-CYSee group.

**Supplementary Figure S3**

**B.**

**A.**

**D.**

**C.**

**Supplementary Figure S3.** Values of EF_50_ (**Panel A**), relaxation time (**Panel B**), apneic pause (**Panel C**) and expiratory delay (**Panel D**) in freely-moving rats prior to (Pre) and following injection of morphine (10 mg/kg, IV) and subsequent injections of vehicle (saline, IV) or D-cysteine ethyl ester (D-CYSee, 500 μmol/kg, IV). The data are presented as mean ± SEM. There were 5 rats in the vehicle group and 6 rats in the D-CYSee group.

**Supplementary Figure S4**

**A.**

**B.**

**Supplementary Figure S4.** Values of non-eupneic breathing index (NEBI) (**Panel A**) and values of NEBI corrected for frequency of breathing (**Panel B**) in freely-moving rats prior to (Pre) and following injection of morphine (10 mg/kg, IV) and subsequent injections of vehicle (saline, IV) or D-cysteine ethyl ester (D-CYSee, 500 μmol/kg, IV). The data are presented as mean ± SEM. There were 5 rats in the vehicle group and 6 rats in the D-CYSee group.

**Supplementary Figure S5**

**A.**

**B.**

**C.**

**Supplementary Figure S5.** Values of frequency of breathing (**Panel A**), tidal volume (**Panel B**) and minute ventilation (**Panel C**) in freely-moving rats prior to (Pre) and following injection of morphine (10 mg/kg, IV) and subsequent injections of vehicle (saline, IV) or D-cysteine (500 μmol/kg, IV). Data are presented as mean ± SEM. There were 5 rats in the vehicle group and 6 rats in the D-cysteine group. Pre-values for Freq in the vehicle and D-cysteine group were 109 ± 4 and 105 ± 3 breaths/min, respectively. Pre-values for TV in vehicle and D-cysteine rats were 2.56 ± 0.12 and 2.50 ± 0.13 ml, respectively. Pre-values for MV in the vehicle and D-cysteine rats were 277 ± 11 and 266 ± 13 ml/min, respectively (P > 0.05, for all between group comparisons).
